# Planar cell polarity protein Dishevelled 3 (Dvl3) regulates ectoplasmic specialization (ES) dynamics in the testis through changes in cytoskeletal organization

**DOI:** 10.1038/s41419-019-1394-7

**Published:** 2019-02-26

**Authors:** Linxi Li, Baiping Mao, Ming Yan, Siwen Wu, Renshan Ge, Qingquan Lian, C. Yan Cheng

**Affiliations:** 1 The Second Affiliated Hospital and Yuying Children’s Hospital, Wenzhou Medical University, Wenzhou, Zhejiang 325027 China; 2 The Mary M. Wohlford Laboratory for Male Contraceptive Research, Center for Biomedical Research, Population Council, 1230 York Ave, New York, 10065 USA; 3 Jiangsu Key Laboratory of Drug Screening, China Pharmaceutical University, Nanjing, 210009 China

## Abstract

In the mammalian testes, such as in rats, the directional alignment of polarized elongating/elongated spermatids, in particular step 17–19 spermatids, across the plane of seminiferous epithelium resembles planar cell polarity (PCP) found in hair cells of the cochlea. It is obvious that spermatid PCP is necessary to support the simultaneous development of maximal number of elongating/elongated spermatids to sustain the daily production of > 50 million sperm per adult rat. Studies have shown that the testis indeed expresses multiple PCP proteins necessary to support spermatid PCP. Herein, using physiological and biochemical assays, and morphological analysis, and with the technique of RNA interference (RNAi) to knockdown PCP protein Dishevelled (Dvl) 1 (Dvl1), Dvl2, Dvl3, or Dvl1/2/3, Dvl proteins, in particular Dvl3, it was shown that Dvl3 played a crucial role of support Sertoli cell tight junction (TJ)-permeability barrier function through changes in the organization of actin- and microtubule (MT)-based cytoskeletons. More important, an in vivo knockdown of Dvl1/2/3 in the testis, defects of spermatid polarity were remarkably noted across the seminiferous epithelium, concomitant with defects of spermatid adhesion and spermatid transport, leading to considerably defects in spermatogenesis. More important, Dvl1/2/3 triple knockdown in the testis also impeded the organization of actin- and MT-based cytoskeletons owing to disruptive spatial expression of actin- and MT-regulatory proteins. In summary, PCP Dishevelled proteins, in particular, Dvl3 is a regulator of Sertoli cell blood–testis barrier (BTB)  and also spermatid PCP function through its effects on the actin- and MT-based cytoskeletons in Sertoli cells.

## Introduction

During spermatogenesis, developing step 17–19 spermatids in the rat testis displays conspicuous planar cell polarity (PCP)^1^. It is noted that the alignment of polarized developing spermatids in stage V–VIII tubules, with their heads and tails point towards the basement membrane and seminiferous tubule lumen, respectively, across the plane of the seminiferous epithelium^1,2^, resembles the directional alignment of hair from hair cells of the inner ear in mammals known as PCP^3–5^. This unusual alignment of developing spermatids across the epithelium thus packs the maximum number of spermatids in a restricted surface area of the epithelium to support the production of millions of sperm on a daily basis from an adult male^2,6^. As such, the fixed population of Sertoli cells in adult testes^7^ can nurture the simultaneous development of millions of germ cells with a Sertoli:germ cell ratio of ~1:30–1:50^8^. It is also necessary to provide orderly interactions between Sertoli cells and spermatids in the microenvironment of the epithelium behind the blood–testis barrier (BTB) to support the developing germ cells structurally, functionally, and nutritionally^2,6,9^. Studies have shown that the testis is equipped with multiple PCP proteins necessary to confer spermatid PCP, such as the PCP core proteins Van Gogh-like (Vangl) proteins (e.g., Vangl2), Dishevelled (Dvl) (e.g., Dvl2, Dvl3), and Frizzled (Fzd) class receptors (e.g., Fzd3, Fzd5)^10^. It is now established that PCP protein Vangl2 is necessary to support spermatogenesis through its regulatory effects on actin- and microtubule (MT)-based cytoskeletons^10^. More important, Vangl2 knockdown in the testis in vivo was found to perturb spermatogenesis considerably, including spermatid exfoliation, but also unwanted retentions of spermatid 19 spermatids in post-stage VIII tubules as spermatid 19 spermatids were found in the epithelium together with step 9, 10, and 11 spermatids in stage IX, X, and XI tubules^10^. Studies from other animal models (in particular insects, worms, and flies) and epithelia have shown that Vangl2/Prickle and Fzd/Dvl are two primary PCP protein complexes wherein Vangl2 and Fzd are integral membrane proteins whereas Prickle and Dvl are the corresponding primary adaptor proteins; and these two PCP protein complexes are mutually exclusive regarding their distribution and functionally^11–14^. To better understand the role of PCP proteins in spermatogenesis, we reported herein results of a series of experiments that delineated the role of Dvl3 (i.e., the adaptor proteins of the integral membrane protein family Fzd) in the testis. The selection of Dvl3 instead of Dvl1 and Dvl2 for more detailed analysis was based on initial observations that its knockdown by RNAi led to considerably more disruptive effects on the Sertoli cell TJ-barrier function compared to Dvl1 and Dvl2. However, for our in vivo studies, Dvl1/2/3 were simultaneously silenced by RNAi to confirm changes in phenotypes, correlating the function of Dvl to support spermatogenesis.

## Materials and methods

### Animals

Adult Sprague–Dawley rats at 250–275 gm b.w. and male pups at 16 days of age were obtained from Charles River Laboratories (Kingston, NY). Adult rats were housed in groups of two in the same cage, and 10 male pups will be housed with a foster mother in the same cage at the Rockefeller University Comparative Bioscience Center with free access to water and standard rat chow and water ad libitum under controlled temperature (22 °C) and constant light–dark cycles (12 h of light and 12 h of darkness). The use of animals and recombinant DNA materials including siRNA duplexes for both in vitro and in vivo experiments reported herein was approved by the Rockefeller University Institutional Animal Care and Use Committee (IACUC) with Protocol Numbers 15–780-H and 18-043-H and Rockefeller University Institutional Biosafety Committee (IBC) with Protocol Number 2-15-04-007, respectively. Rats were killed by CO_2_ asphyxiation using slow (20–30%/min) displacement of chamber air from compressed carbon dioxide in an euthanasia chamber built and approved by the Rockefeller University Laboratory Safety and Environmental Health.

### Antibodies, primer sequences, and siRNA duplexes

Antibodies used for various experiments reported herein were obtained commercially unless otherwise specified (Table 1), and these antibodies have been characterized as noted in our earlier reports and known to cross-react with the corresponding proteins in rats as indicated by the manufacturers (or earlier by us in published reports). The RRID (Research Resource Identifier) information and working dilutions for different applications for various experiments in this report are also listed in Table 1. Information on primer pairs and siRNA duplexes are listed in Tables 2 and 3, respectively.

**Table 1:** Antibodies used for different experiments in this report

Antibody (RRID No.)	Host species	Vendor	Catalog number	Working dilution	
				IB	IF
Dvl3 (AB_92182)	Rabbit	Millipore	AB5974		1:200 (SC)
Dvl3 (AB_2230636)	Rabbit	Cell Signaling Technology	3218	1:1000	
Dvl3 (AB_1841289)	Rabbit	Sigma-Aldrich	WH0001857M4		1:250 (T)
Dvl1 (AB_627430)	Mouse	Santa Cruz Biotechnology	sc-8025	1:500	
Dvl2 (AB_627432)	Mouse	Santa Cruz Biotechnology	sc-8026	1:500	
Fzd3 (AB_10988754)	Mouse	Santa Cruz Biotechnology	sc-376105	1:500	
INVS (AB_2233902)	Rabbit	Protein Tech	10585-a-AP	1:500	
Vangl2 (AB_2213082)	Goat	Santa Cruz Biotechnology	sc-46561	1:500	
CAR (AB_2087557)	Rabbit	Santa Cruz Biotechnology	sc-15405	1:200	1:50
ZO-1 (AB_2533938)	Rabbit	Thermo Fisher Scientific	61–7300	1:250	1:100
N-cadherin (AB_647794)	Rabbit	Santa Cruz Biotechnology	sc-7939	1:200	
N-cadherin (AB_2313779)	Mouse	Thermo Fisher Scientific	33–3900		1:100
β-catenin (AB_634603)	Rabbit	Santa Cruz Biotechnology	sc-7199	1:250	
β-catenin (AB_2533982)	Rabbit	Thermo Fisher Scientific	71–2700		1:100
Eps8 (AB_397544)	Mouse	Thermo Fisher Scientific	610143	1:5000	1:100
Formin1 (AB_2105244)	Mouse	Abcam	ab68058	1:500	
Arp3 (AB_476749)	Mouse	Sigma-Aldrich	A5979	1:3000	1:100
MARK4 (AB_2140610)	Rabbit	Cell Signaling Technology	4834	1:500	
EB1 (AB_2141629)	Rabbit	Santa Cruz Biotechnology	sc-15347	1:200	1:300
Dia1 (AB_2092924)	Goat	Santa Cruz Biotechnology	sc-10885	1:200	
Clip170 (AB_2082238)	Rabbit	Santa Cruz Biotechnology	sc-25613	1:200	
α-tubulin (AB_2241126)	Mouse	Abcam	ab7291	1:1000	1:200
β-tubulin (AB_2210370)	Rabbit	Abcam	ab6046	1:1000	
Detyrosinated α-tubulin (AB_869990)	Rabbit	Abcam	ab48389	1:1000	1:200
Acetylated α-Tubulin (AB_448182)	Mouse	Abcam	ab24610	1:1000	
Tyrosinated α-Tubulin (AB_261811)	Mouse	Sigma-Aldrich	T9028	1:1000	
Vimentin (AB_628437)	Mouse	Santa Cruz Biotechnology	sc-6260	1:200	
β-Actin (AB_2714189)	Mouse	Santa Cruz Biotechnology	sc-47778	1:500	
GAPDH (AB_2107448)	Mouse	Abcam	ab8245	1:1000	
Claudin 11 (AB_2533259)	Rabbit	Thermo Fisher Scientific	36–4500	1:250	
Occludin (AB_2533977)	Rabbit	Thermo Fisher Scientific	71–1500	1:250	
Goat anti-Rabbit IgG-HRP (AB_2534776)	Goat	Thermo Fisher Scientific	A16104	1:20000	
Goat anti-Mouse IgG-HRP (AB_2534745)	Goat	Thermo Fisher Scientific	A16072	1:10000	
Bovine anti-Goat IgG-HRP (AB_634811)	Bovine	Santa Cruz Biotechnology	sc-2350	1:3000	
Rabbit IgG-Alexa Fluor 488 (AB_2576217)	Goat	Thermo Fisher Scientific	A-11034		1:250
Mouse IgG-Alexa Fluor 488 (AB_2534088)	Goat	Thermo Fisher Scientific	A-11029		1:250
Rabbit IgG-Alexa Fluor 555 (AB_141784)	Goat	Thermo Fisher Scientific	A-21428		1:250
Mouse IgG-Alexa Fluor 555 (AB_141780)	Goat	Thermo Fisher Scientific	A-21424		1:250

Arp3, actin-related protein 3, which together with Arp2 create the Arp2/3 complex known to induced branched actin polymerization, converting linear actin filaments into a branched network; CAR, coxsackievirus and adenovirus receptor, a TJ integral membrane protein; Clip170, cytoplasmic linker protein 170 α1, a microtubule plus (+)-end tracking protein, +TIP; Dia1, diaphanous homolog 1, required for the assembly of F-actin; Dvl3, Dishevelled 3; EB1, end-binding 1 protein, a microtubule plus (+)-end tracking protein, or +TIP; Eps8, epidermal growth factor receptor pathway substrate 8, an actin barbed end capping and bundling protein; Fzd3, Frizzled 3; INVS, inversin; GAPDH, glyceraldehyde 3-phosphate dehydrogenase; Vangl2, Van Gogh 2, a PCP protein; ZO-1, zonula occludens-1

**Table 2:** Primers used for RT-PCR

Gene	Accession number	Orientation	Nucleotide sequence	Nucleotide position	Product length (BP)
*Dvl1*	NM_031820.1	Sense	5′-CCACAGCTTGAGGAGGCA-3′	1204–1221	313
		Anti-Sense	5′-ATGCGAGGTTGCTGCACA-3′	1499–1516	
*Dvl2*	NM_001172056.1	Sense	5′-GGCTGCGAGAGTTACCTA-3′	1531–1548	320
		Anti-Sense	5′-CCAGACTTTGACTCAGGG-3′	1833–1850	
*Dvl3*	NM_001107081.2	Sense	5′-CCATCACCAGCTCCATCC-3′	1025–1042	667
		Anti-Sense	5′-CTGCTGCGTGTAGTGTGG-3′	1674–1691	
*S16*	NM_001169146.1	Sense	5′-TCCGCTGCAGTCCGTTCAAGTCTT-3′	87–110	385
		Anti-Sense	5′-GCCAAACTTCTTGGATTCGCAGCG-3′	448–471

**Table 3:** siRNA duplexes used for RNAi experiments

Gene	Product	Catalog number	Target sequences (5′-3′)
*Dvl1*	ON-TARGETplus rat Dvl1 (83721) siRNA-SMARTpool	L-096590-02-0020	CCUCUCGCUAAUUCGUAA
			AGGAGAUCUUCGACGACAA
			UGAGAGGACAGGCGGCAUU
			AGCGAAGGGAGGCGAGGAA
*Dvl2*	ON-TARGETplus rat Dvl2 (303251) siRNA-SMARTpool	L-083872-00-0020	GGGCCAAAGUAACGAGCGU
			GAGGAUGACAGCAUGAGUA
			GGCGAUUUCAAGAGCGUUU
			GUGGUGUAGGCGAGACGAA
*Dvl3*	ON-TARGETplus rat Dvl3 (303811) siRNA-SMARTpool	L-081790-00-0020	CAUGAGUAAUGAUGACGCA
			GCACAGGAGGCAUCGGUGA
			GAGCAGUGCCUCACGCCUA
			UGGUAGCGGCAGCGAGUCA
Non-targeting negative control	ON-TARGETplus Non-targeting siRNA duplexes pool	D-001810-10-0050	UGGUUUACAUGUCGACUAA
			UGGUUUACAUGUUGUGUGA
			UGGUUUACAUGUUUUCUGA
			UGGUUUACAUGUUUUCCUA

### Primary cultures of Sertoli cells

Sertoli cells were isolated from testes of male pups at 20 days of age as described^15^. Freshly isolated Sertoli cells suspended in Ham's F12 Nutrient Mixture (F12)/Dulbecco's Modified Eagle's Medium (DMEM) (Sigma-Aldrich), supplemented with growth factors (bovine insulin, human transferrin, epidermal growth factor), bacitracin, and gentamicin were seeded on Matrigel (Fisher Scientific)-coated culture dishes (either 6, 12-, or 24-wells), coverslips (to be placed in 12-well dishes) and bicameral units (Millipore, Billerica, MA) (to be placed in 24-well dishes) at a density 0.4–0.6 × , 0.025–0.04 × , and 1 × 10^6^ cells/cm^2^, respectively, as described^15^. Cells were then placed in a humidified CO_2_-incubator with 95% air/5% CO_2_ (vol/vol) at 35 °C during the experimental period. For 6-well and 12-well dishes, each well contained 5 and 3 ml F12/DMEM medium supplemented with growth factors and antibiotics, respectively, and these Sertoli cells were used for biochemical assays or for immunoblottings (IB). For immunofluorescence analysis (IF), each well (containing coverslip) had 2 ml F12/DMEM. For bicameral units placed in 24-well dishes, the apical and the basal chamber contained 0.5 ml each of F12/DMEM, supplemented with growth factors and gentamicin^15^. These Sertoli cell cultures with minimal Leydig, germ and peritubular myoid cell contaminations were used for experiments on day 3 after a functional tight junction (TJ)-permeability barrier was established. Also, ultrastructures of actin-based TJ, basal ES, and gap junction, as well as intermediate filament-based desmosome that mimicked the Sertoli cell BTB in vivo were also detected when examined by electron microscopy as described^16–18^.

### Isolation of germ cells

Total germ cells, including spermatogonia, spermatocytes, round spermatids, elongating spermatids, and elongated spermatids were isolated from testes of adult Sprague–Dawley rats (~ 300 gm b.w.) as detailed elsewhere^19^. The purity of these germ cell preparations had been validated and also characterized in our laboratory^20^, including DNA flow cytometric analysis, illustrating the relative ratio of different germ cells types obtained with this procedure mimicked those found in the testis^19^. These germ cells were used for RNA extraction within 6 h following their isolation.

### Knockdown of Dvl3 vs. Dvl1/2/3 by RNA interference (RNAi) in Sertoli cells in vitro

Dvl3 was selected for in vitro silencing experiments as reported herein since pilot experiments had shown that its knockdown (vs. Dvl1 or  Dvl2) caused the most severe disruption of the Sertoli TJ-permeability barrier. In short, Dvl3 RNAi was performed by using siRNA duplexes specific to Dvl3 and also Dvl1 and Dvl2 vs. Dvl1/2/3 (Table 3) for triple knockdown. In brief, Sertoli cells were cultured alone for 3 days after a functional TJ-permeability barrier was established when the transepithelial electrical resistance (TER) across the Sertoli cell epithelium had reached its plateau. On day 3, Sertoli cells were transfected with corresponding Dvl-specific small interfering RNA (siRNA) duplexes vs. non-targeting negative control (Dharmacon) duplexes at 50 nm (for RT-PCR, IB, actin polymerization/bundling assay, IF and TJ-permeability barrier integrity assay) using RNAiMAX (Life Technologies) as a transfection reagent for 12 h as earlier described^21,22^ (Table 3). Thereafter, cells were washed thrice with fresh F12/DMEM to remove transfection reagents, replaced with fresh F12/DMEM and cultured for an additional 48 h (that is, day 5.5) before RNA was extracted for analysis by RT-PCR, lysates were obtained for IB, actin and MT polymerization assays, or Sertoli cells were used for IF. For assessment of the Sertoli cell TJ-barrier function, TER reading was taken 12 h after transfection and then daily until day 7. For cultures to be used for IF, cells were co-transfected with 1 nm siGLO red transfection indicator (red fluorescence,  Dharmacon) to track successful transfection. In each experiment, replicates or triplicates were used for each treatment vs. control groups, and each experiment was repeated with *n* = 3 using different batches of Sertoli cells, which yielded similar reagent.

### Knockdown of Dvl1/2/3 in adult rat testes in vivo

Since pilot experiments by silencing Dvl3 alone did not yield notably phenotypes in the testis in vivo, possibility due to the presence of Dvl1 and Dvl2 that could supersede (or compensate, at least in part)  the lost function of Dvl3, a triple knockdown of Dvl1/2/3 in the testis using corresponding siRNA duplexes (Table 3) was performed. In brief, Dvl1/2/3 was silenced in the testis of adult rats by transfecting testes with Dvl1/2/3 siRNA duplexes vs. non-targeting control siRNA duplexes using Polyplus in vivo-jetPEI (Polyplus transfection S.A., Illkirch, France) as a transfection reagent according to manufacturer’s instructions^23,24^. Studies from our laboratory had shown that this transfection reagent had a considerable improvement in transfection efficiency (at ~ 70%) vs. conventional transfection medium (at ~ 30%)^22,24–26^. In brief, siRNA duplexes (600 nm with 200 nm each of Dvl1, Dvl2 and Dvl3 vs. non-targeting negative control siRNA duplexes of 600 nm) were constituted in 100 µl of transfection solution containing 1.7 µl in vivo-jetPEI (an adult rat testis was at ~ 1.6 g in weight, with a volume of ~ 1.6 ml) according to manufacturer’s recommendations with N/P = 8 (note: N/P ratio refers to the number of nitrogen (N) residues of jetPEI/nucleotide phosphate (P), and jetPEI concentration is expressed in nitrogen residues molarity and 1 µg of plasmid DNA contains 3 nmol of anionic phosphate; i.e., 600  nm ( ~12  µg) siRNA required 1.7 µl in vivo-jetPEI). The transfection mixture was administered to each testis using a 28-gauge 13-mm needle attached to a 0.5 ml insulin syringe. The needle was inserted from the apical to the basal end of the testis vertically in which the right testis was transfected with the Dvl1/2/3 siRNA duplexes vs. the left testes transfected with the negative non-targeting control siRNA duplexes. As the needle was withdrawn apically, transfection solution was gently released and filled the entire testis to avoid an acute rise in intra-testicular hydrostatic pressure, which was shown to cause physical damage to the seminiferous epithelium. Transfection was performed on day 0, 2, and 4 (triple transfections, *n* = 3 rats). Rats were killed on day 6 by CO_2_ asphyxiation using slow (20–30%/min) displacement of chamber air from a carbon dioxide tank. Testes were removed immediately, frozen in liquid nitrogen or fixed in Bouin’s fixative or modified Davidson’s fixative^27,28^ for their subsequent use as described^22^.

### Monitoring Sertoli cell TJ-permeability barrier function in vitro

The TJ-permeability barrier function across the Sertoli cell epithelium when Sertoli cells were cultured on Millipore bicameral units (diameter 12 mm; pore size 0.45 µm, effective surface area 0.6 cm^2^; EMD Millipore) at 1.0 × 10^6^ cells/cm^2^ was performed as earlier described^15^. Each bicameral unit was placed inside the well of a 24-well dish with 0.5 ml F12/DMEM each in the apical and the basal compartments. Dvl1, Dvl2, Dvl3, or Dvl1/2/3 (vs. control) was silenced in Sertoli cells cultured alone on day 3 by RNAi using specific siRNA vs. non-targeting negative control duplexes at 50 nm (for Dvl1/2/3 triple knockdown, 50 nm Dvl was used to a total of 150 nm) for 12 h, and Sertoli cell TJ-permeability barrier function was monitored daily by quantifying TER across the cell epithelium^15^. In each experiment, each treatment and the control group had triplicate or quadruple bicameral units, with a total of *n* = 3 independent experiments using different batches of Sertoli cells.

### RNA extraction and RT-PCR

Total RNA was isolated from rat testes, Sertoli cells, germ cells, small intestine, and liver using Trizol reagent (Life Technologies) and RT-PCR was performed as described^10^. A total of 2 µg RNA was reverse transcribed by Moloney murine leukemia virus reverse transcriptase (Promega, Madison, WI) according to manufacturer’s instructions to obtain complementary DNAs (cDNAs), which served as templates for subsequent PCR using primer pairs specific to Dvl1, Dvl2, or Dvl3 vs. S16, which served as the PCR loading control (Table 2). Authenticity of PCR products was verified by gel electrophoresis, which was then extracted for direct DNA sequencing at Genewiz (South Plain field, NJ). Each RT-PCR experiment was performed with *n* *=* 3 experiments, which yielded similar results. RT-PCR was also used to confirm specific knockdown of Dvl1, Dvl2, or Dvl3.

### IB (immunoblot)  analysis

Lysates of Sertoli cells or testes were obtained using lysis buffer (50 mm Tris, containing 0.15 m NaCl, 1% Nonidet P-40 (vol/vol), 1 mm ethylene glycol-bis(β-aminoethyl ether)-*N*,*N*,*N*′,*N*′-tetraacetic acid (EGTA), 10% glycerol (vol/vol), pH 7.4 at 22 °C, freshly supplemented with protease inhibitor mixture (Sigma-Aldrich) and phosphatase inhibitor cocktail II (Sigma-Aldrich)) for IB analysis as described^10^. Protein estimation was performed using BSA as a standard with a Bio-Rad DC protein assay kit (Bio-Rad Laboratories, Hercules, CA). IB analysis was performed using equal amount of total protein lysate between samples in each experiment at 20 μg or 60 μg protein for cell or testis lysates, respectively, with corresponding antibodies for different marker proteins (Table 1) and in-house prepared chemiluminescence kits as described^15^. Chemiluminescence signals were detected using an ImageQuant LAS 4000 (GE Healthcare Life Sciences) Imaging system and ImageQuant software (Version 1.3). Glyceraldehyde 3-phosphate dehydrogenase (GAPDH)  or β-actin served as a protein loading control. Protein band intensities were evaluated by ImageJ 1.45 s software obtained at http://rsbweb.nih.gov/ij (National Institutes of Health (NIH), Bethesda, MD). All samples within an experimental group were processed simultaneously to avoid inter-experimental variations. Each sample had triplcates in both silencing and control groups with *n* = 3 independent experiments using different batches of Sertoli cells or testes from different rats.

### Histological, IF (immunofluorescence), F-actin staining, and fluorescence image analysis

Histology was performed using cross-sections of testes fixed in modified Davidson fixative, embedded in paraffin and cut with a microtome (5 µm in thickness) for H&E (hematoxylin and eosin) staining as described^29^. IF was performed using frozen cross-sections of testes at 7 μm obtained in a cryostat at −22 °C, or Sertoli cells cultured on coverslips as described^21,29,30^. Target proteins were visualized by incubating testis sections or cells with a specific primary and the corresponding secondary antibodies (Table 1), and co-stained with 4′,6-diamidino-2-phenylindole (DAPI)  (Sigma) to visualize cell nuclei. Slides were mounted in Prolong Gold Antifade reagent (Invitrogen, Life Technologies). For F-actin staining, sections or Sertoli cells were incubated with Alexa Fluor 488 phalloidin (Invitrogen) according to manufacturer’s instructions as described^22^. Images were examined and acquired using a Nikon Eclipse 90i Fluorescence Microscope system equipped with Nikon Ds-Qi1Mc or DsFi1 digital camera and Nikon NIS Elements AR 3.2 software (Nikon, Tokyo, Japan) and saved in TIFF format. Image overlays were performed using Adobe Photoshop CS4 (San Jose, CA). Fluorescence intensity was analyzed by ImageJ 1.45 s (NIH, Bethesda, MD) or Nikon NIS Elements AR (Version 3.2) software package. All samples from control and treatment groups were analyzed in a single experimental session to avoid inter-experimental variations. Data shown here were representative data from a single experiment, with a total of *n* = 3 independent experiments. For fluorescence intensity or distribution analysis in Sertoli cells or seminiferous tubules of testes, at least 100 cells or 100 cross-sections of testes were randomly selected and examined in both experimental and control groups, and a total *n* = 3 experiments were performed.

### Assessing the ratio of F (filamentous) actin to G (globular) actin by a spin-down assay

The relative ratio of F:G-actin in Sertoli cell or testis lysates following Dvl1, 2, 3, or Dvl1/2/3 knockdown was assessed according to the manufacturer’s instructions (Cat No. BK037, Cytoskeleton, Denver, CO) to assess the ability of these cell lysates to polymerize actin monomers into F-actin as described^22^. Samples treated with phalloidin (0.1 µM, actin stabilizing agent) vs. urea (80 mM, actin depolymerization agent) served as the corresponding positive and negative controls.

### Actin polymerization assay

Actin polymerization assay to assess the ability of Sertoli cell lysates following Dvl silencing vs. controls to polymerize pyrene actin oligomers was performed as described^21,31^ according to manufacturer’s instructions (Cat No. BK003, Cytoskeleton). Polymerization and polymerization kinetics were monitored in a Corning 96-well black flat bottom polystyrene microplate (Corning, Lowell, MA, USA) using a FilterMax F5 Multi-Mode Microplate Reader from the top and the Multi-Mode Analysis Software 3.4 (Molecular Devices, Sunnyvale, CA) at room temperature (fluorimeter settings, measurement type: kinetic, 100 cycle, 20 sec interval; excitation wavelength: 360 nm; emission wavelength: 430 nm; integration time: 0.25 ms). Actin polymerization rate (i.e., kinetics) that monitored the polymerization of pyrene-labeled actin was monitored by an increase in fluorescence intensity, and the rate of actin polymerization (i.e., an increase in fluorescence intensity over time) during the initial linear phase in the first 10 min was estimated by linear regression analysis using Microsoft Excel 2016 (Microsoft, WA). Phalloidin (1 µm, an actin stabilizing agent) vs. urea (100 mm, an actin depolymerization agent) recommended by the manufacturer was used as the corresponding positive and negative controls. For actin assays, the same amount of Sertoli cell lysate (40 µg total protein) or testis lysate (60 µg total protein) from each sample was also analyzed by immunoblot to confirm the knockdown of Dvl1, 2, or 3 vs. Dvl1/2/3. Each sample had replicate dishes with *n* *=* 3 independent experiments.

### MT spin-down assay to assess MT polymerization

MT spin-down assay was performed as described^32,33^. This assay estimated the relative level of polymerized MTs vs. free tubulins in Sertoli cell cytosol according to the manufacturer’s protocols (Cat No. BK038, Cytoskeleton, Denver, CO). Paclitaxel (20 µm, also known as Taxol, a MT stabilizing agent) vs. CaCl_2_ (2 mm, a MT depolymerization agent) was used as the corresponding positive and negative controls by including either paclitaxel or CaCl_2_ in the Sertoli cell lysate. In short, this assay assessed changes in the relative distribution of MTs/polymerized tubulins vs. free/non-polymerized tubulin monomers in the pellet vs. supernatant (S/N), respectively, following Dvl3 knockdown when compared with control cells transfected with non-targeting negative control siRNA duplexes.

### Statistical analysis

Statistical analysis was performed with GraphPad Prism 6 software (GraphPad Software) using either Student *t* test (for two-group comparisons), one-way analysis of variance (ANOVA) (for multi-group comparisons), or two-way ANOVA with Bonferroni post hoc tests. All experiments had three to five replicate samples with a total of at least *n* = 3 experiments for analysis.

## Results

### Expression of Dishevelled 3 (Dvl3) in Sertoli cells and its stage-specific localization in the seminiferous epithelium of adult rat testes

**Fig. 1:**
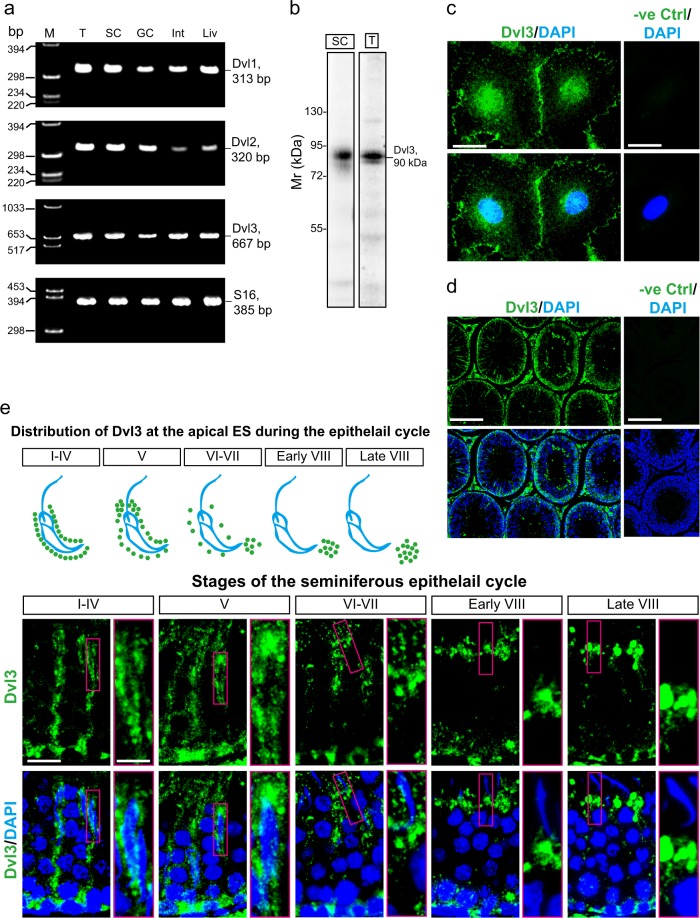
Cellular and stage-specific distribution of Dvl3 during the seminiferous epithelial cycle in adult rat testes. **a** Using mRNAs extracted from testes (T) of adult rats, Sertoli cells (SC) from 20-day-old rats (day 4 cultures), germ cells (GC) freshly isolated from adult rat testes, small intestines (Int), and liver (Liv) from adult rats for RT-PCR with the corresponding primer pairs specific for Dvl1, Dvl2, Dvl3 (Table 2), all three Dishevelled mRNAs were found to be expressed by Sertoli and germ cells in the testis. Representative data from a single experiment, and *n* *=* 3 independent experiments yielded similar results. **b** Immunoblot analysis illustrating the specificity of the anti-Dvl3 antibody (Table 1), which detected a prominent band of 90 kDa using lysates (40 µg protein per lane) of either Sertoli cells (SC) or testes (T), consistent with the Mr of Dvl3 as earlier reported^34^. **c** Dvl3 (green fluorescence) distribution in Sertoli cells cultured in vitro for 3 days was shown herein, which localized prominently to the Sertoli cell–cell interface, with some Dvl3 staining in cytosol and cell nuclei, consistent with its localization in cell cortical zone, cytosol, and cell nuclei as shown in other mammalian cells as  earlier reports^34, 35^. No Dvl3 staining was noted when the primary antibody was substituted by normal rabbit IgG (negative control), illustrating the specificity of staining for Dvl3 shown in micrographs in the left panel. Scale bar, 40 µm, which applies to other micrographs. **d** Localization of Dvl3 in cross-sections of testes, illustrating possible stage-specific expression of Dvl3 in the seminiferous epithelium of adult rat testes. No Dvl3 staining was found in control sections in which the primary antibody was substituted with the corresponding normal rabbit IgG, illustrating staining specificity. Scale bar, 400 µm, which applies to other micrograph in the same panel. **e** Schematic drawing in the upper panel, illustrating stage-specific localization of Dvl3 at the apical ES during the epithelial cycle, which was prepared based on the observations noted in the images in the lower panel. Fluorescent images regarding the distribution of Dvl3 (green fluorescence) across the seminiferous epithelium in representative stages of the epithelial cycle, in particular the enlarged boxed rectangles in red, illustrating changes in Dvl3 distribution at the apical ES. In brief, Dvl3 prominently expressed around the entire spermatid heads at the apical ES in stage I–IV and V tubules. In stage VI–VII, Dvl3 began to move away from the spermatid head, more concentrated to the tip of spermatid heads, and Dvl3 no longer wrapped around the spermatid head in stage VIII tubules, apparently being phagocytosed (or endocytosed) by Sertoli cells, appearing as droplets inside the Sertoli cell. Scale bar, 80 µm; 40 µm in inset, which apply to corresponding micrographs and insets

Using primer pairs specific to Dvl1, 2, and 3 vs. S16 in rats for RT-PCR (Table 2), all Dvls were shown to be expressed in the testis (T), Sertoli cells (SC), and germ cells (GC), in which cDNAs obtained from small intestine (Int) and liver (Liv) served as the positive controls (Fig. 1a). S16 served as a loading and internal PCR control (Fig. 1a). We next used immunoblotting to assess the specificity of the anti-Dvl3 antibody (Table 1) using lysates from Sertoli cells (SC) and adult rat testes (T) (with 40 µg protein per lane), confirming the specificity of this anti-Dvl3 antibody as only a prominent band corresponding to the Mr of Dvl3 at 90 kDa (Fig. 1b) as reported^34^. This antibody (Table 1), selected from four antibodies from different vendors, was next used to examine its cellular localization in Sertoli cell epithelium cultured in vitro for 3 days. Consistent with reports in other epithelia^34,35^, Dvl3 (green fluorescence) was found to localize prominently at the Sertoli cell–cell interface, but some also found in cell cytosol and the cell nuclei (Fig. 1c). No staining was noted in negative control cells wherein the primary antibody was substituted with normal rabbit IgG at the same dilution as of the anti-Dvl3 antibody, illustrating the specificity of the staining (Fig. 1c). In cross-sections of testes, Dvl3 was found near the base of the seminiferous epithelium, but also appeared as track-like structures and also associated with developing spermatids, displaying distinctive stage-specific distribution in the epithelium, but no staining in the negative control sections (Fig. 1d). A careful examination of Dvl3 in the seminiferous epithelium had noted that Dvl3 appeared as track-like structures mostly in stages I–V tubules, but gradually diminished in VI–VII and virtually undetected in VIII tubules and thereafter (Fig. 1e). However, Dvl3 stained prominently at the basal region of the epithelium, consistent with its localization at the basal ES/BTB at all stages of the epithelial cycle  (Fig. 1e). Interestingly, its localization at the apical ES was considerably shifted at the apical ES during spermiogenesis (see upper panel in Fig. 1e vs. enlarged images in lower panel in Fig. 1e). In early stages of the epithelial cycle, Dvl3 was found to surround the entire spermatid heads in stage I–V, as spermiogenesis progressed, Dvl3 shifted to the tip of spermatid heads, and eventually moved away from the spermatid head, possibly being phagocytosed by Sertoli cells or being endocytosed (perhaps being recycled to maintain PCP for newly formed developing spermatids such as step 8 spermatids) in late stage VIII tubules (Fig. 1e).

### Dvl3 co-localizes with F-actin and MTs in the seminiferous epithelium of rat testes, and its changes in expression using the adjudin model

**Fig. 2:**
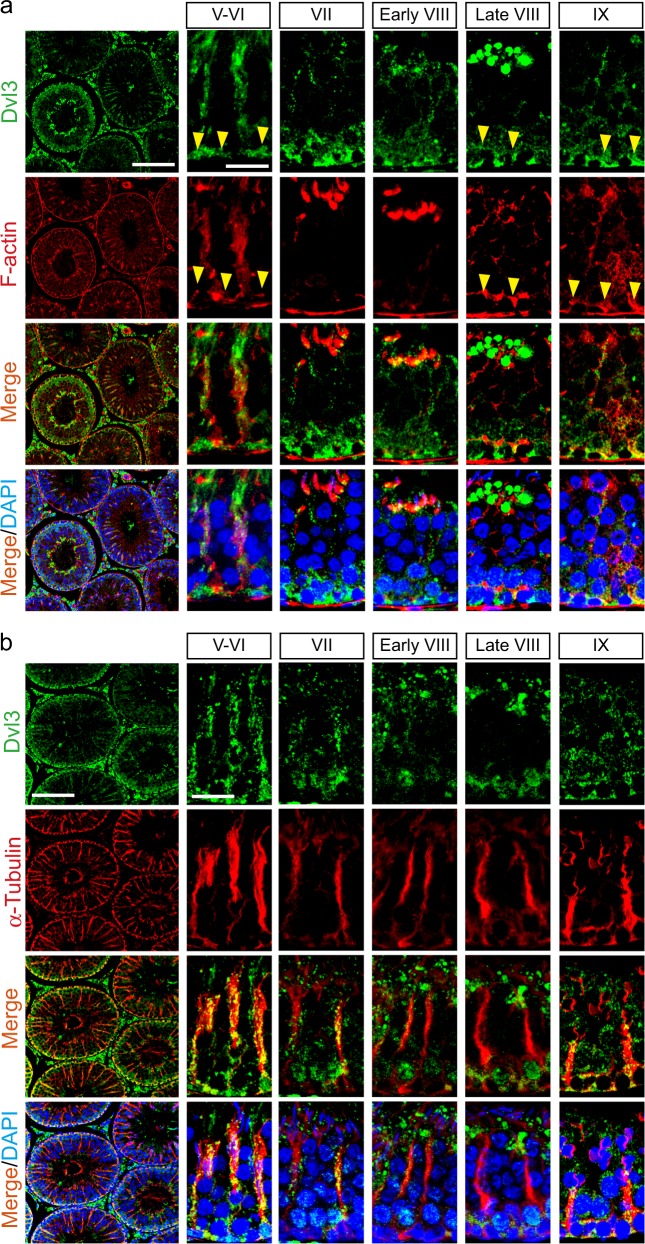
Colocalization of Dvl3 with actin- and MT-based cytoskeletons in the seminiferous epithelium of rat testes. **a** Dvl3 (green fluorescence) was found to colocalize with F-actin (red fluorescence) at the site of the BTB/basal ES (annotated by yellow arrowheads), apical ES, and also track-like structures across the epithelium. Scale bar, 400 µm and 40 µm, which apply to corresponding micrographs in the same panel. **b** Dvl3 (green fluorescence) also colocalized with α-tubulin (red fluorescence; α- together with β-tubulin create the α-/β-tubulin dimers, which are the building blocks of MTs), and the MT-conferred tracks were notably detected in the epithelium, which laid perpendicular to the basement membrane at the base of the seminiferous epithelium. Scale bar, 400 µm and 40 µm, which apply to corresponding micrographs in the same panel

**Fig. 3:**
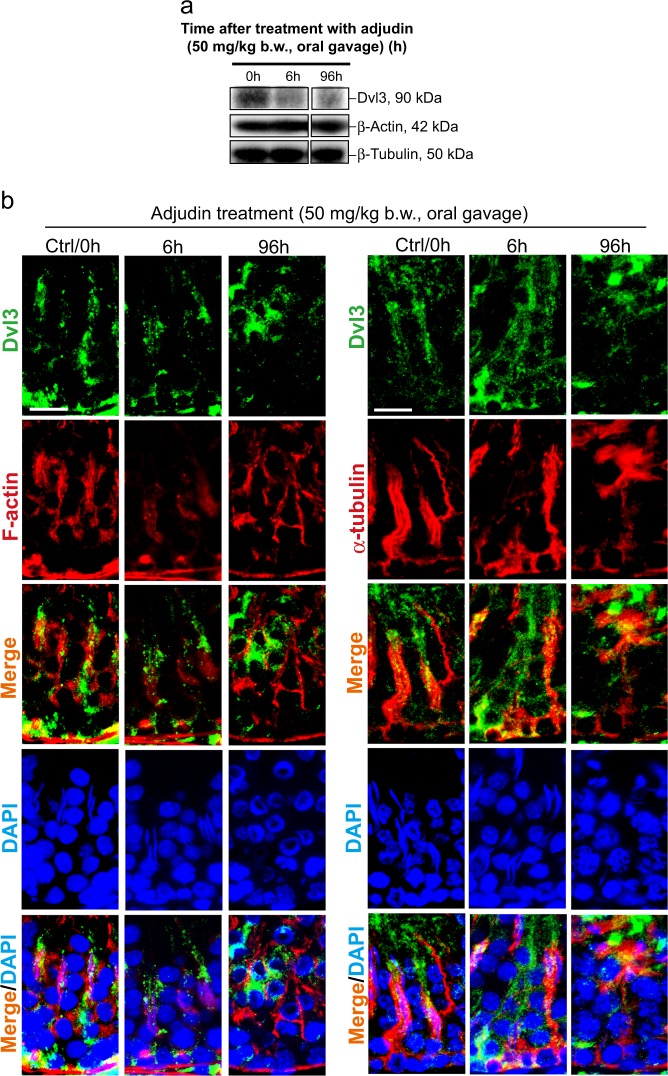
Changes in the expression and distribution of Dvl3 in the testis using the adjudin model. **a** Using the adjudin animal model that is known to mimic the sequence of events leading to the release of sperm at spermiation to study the biology of spermiation^36^, it was shown that adjudin treatment (50 mg/kg b.w., by oral gavage) rapidly downregulated the expression of Dvl3 within 6 h. Uncropped immunoblots (IBs) were shown in Figure S1. **b** Distribution of Dvl3 (green fluorescence) was rapidly perturbed following adjudin treatment, and this disruptive changes in Dvl3 distribution closely mimicked adjudin-induced disruption of the actin (red fluorescence)- and MT (red fluorescence)-based cytoskeletal organization. Following adjudin treatment, the track-like structures conferred by F-actin surrounding the developing spermatids in these stage VI–VII tubules were grossly disrupted; and virtually no elongating/elongated spermatids were noted in the seminiferous epithelium in all the tubules examined by 96 h when compared with control testes. Scale bar, 40 µm, which applies to corresponding micrographs in the same panel

Dvl3 appeared as track-like structures that stretched across the epithelium, and laid perpendicular to the basement membrane, in stages V–VI and VII tubules, and closely associated with developing spermatids (Fig. 2a, b), notably similar to the organization of F-actin (mostly in stage V–VI tubules) (Fig. 2a) and MTs (Fig. 2b), colocalizing with these two cytoskeletons (Fig. 2a, b). Dvl3 was also found at the basal ES/BTB (see yellow arrowheads) colocalizing with F-actin (Fig. 2a) and also α-tubulin (which together with β-tubulin create α-/β-tubulin dimers to serve as the building blocks of MTs) (Fig. 2b). Using the animal model of adjudin by treating adult rats with a single dose of adjudin at 50 mg/kg b.w. (by oral gavage) which is known to induce germ cell exfoliation by disrupting the actin- and MT-based cytoskeletal^36^, the steady-state protein level of Dvl3 was remarkably downregulated by 6 h post treatment and persisted through 96 h when most of the spermatids were depleted from the epithelium (Fig. 3a). Interestingly, adjudin treatment caused extensive disarray on the distribution of Dvl3 across the epithelium when the F-actin and MT organization was also grossly perturbed (Fig. 3b).

### Dvl3 knockdown in Sertoli cells perturbs the TJ-barrier function through disruptive changes on the distribution of TJ- and basal ES proteins at the cell–cell interface

**Fig. 4:**
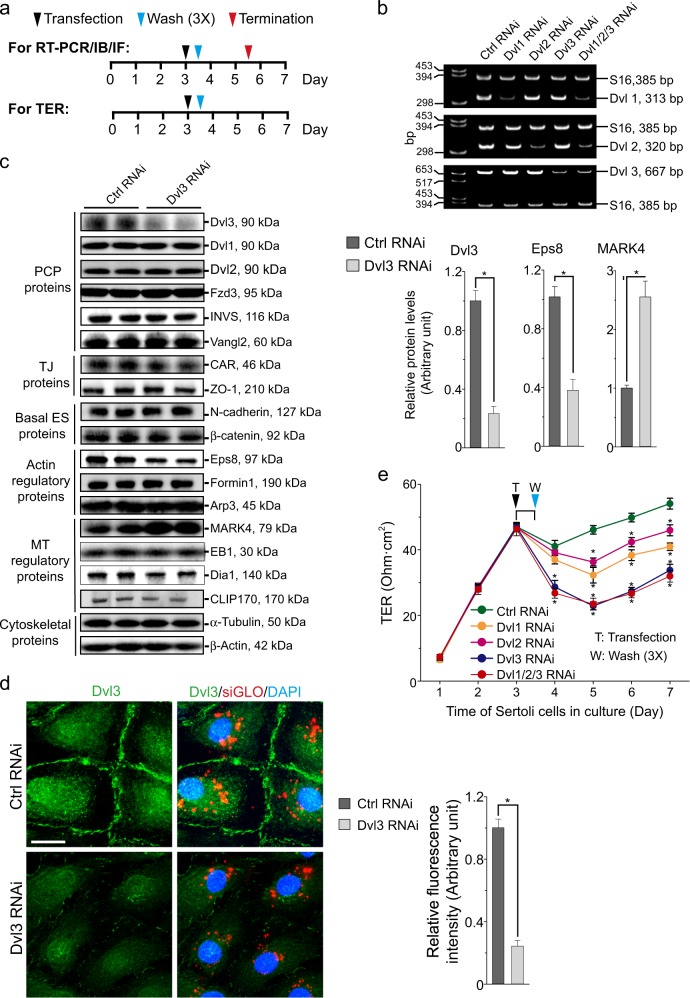
A knockdown of Dvl3 in Sertoli cells cultured in vitro on the TJ-permeability barrier function. **a** Regimen used in this study to monitor the effects of Dvl3 knockdown by RNAi to assess: (i) changes in the steady-state level of target gene expression by RT-PCR or protein level by immunoblotting (IB) and also protein expression by immunofluorescence analysis (IF); or (ii) changes in Sertoli cell TJ-permeability barrier function by quantifying transepithelial resistance (TER) across the Sertoli cell epithelium on the bicameral units. Transfection of Sertoli cells with siRNA duplexes (including negative non-targeting control siRNA duplexes) was performed on day 3 for 12 h prior to their removal by washing (thrice) with F12/DMEM medium, based on pilot experiments. **b** Representative findings of an RT-PCR experiment, illustrating specificity of Dishevelled protein knockdown, in which knockdown of Dvl1, Dvl2, or Dvl3 using corresponding specific siRNA duplexes (Table 2) only downregulated the expression of the intended Dvl gene expression. **c** Representative IB results illustrating a knockdown of Dvl3 by almost ~ 80% (see bar graph on the right panel also) failed to perturb the expression of multiple BTB-associated proteins except a considerably down-regulation of Eps8 (an actin barbed end capping and bundling protein) and up-regulation of MARK4 (a Ser/Thr protein kinase known to stabilize MT^45^) (see also right panel for composite data of *n* = 3 independent experiments), thereby perturbing the corresponding actin- and MT-based cytoskeletal function. Uncropped IBs were shown in Figure S2. Each bar in the histogram shown in the right panel is a mean ± SD of *n* = 3 independent IB experiments. *P < 0.01, compared to the corresponding control by Student’s *t* test. **d** Fluorescent staining of Dvl3 (green fluorescence) at the Sertoli cell–cell interface (and some in cell cytosol and in cell nuclei), illustrating a ~ 80% knockdown of Dvl3 following transfection of Sertoli cells with Dvl3-specific siRNA duplexes vs. non-targeting negative control siRNA duplexes (see also bar graph on the right panel for the composite data). Successful transfection was indicated by siGLO (red fluorescence) transfection reagent. Scale bar, 40 µm, which applies to all other micrographs. Each bar in the bar graph on the right panel is a mean ± SD of *n* = 3 independent experiments. *P < 0.01, by Student’s *t* test. **e** A knockdown of Dvl3 (vs. Dvl1 and Dvl2 when compared with control) was considerably more effective to perturb the Sertoli cell TJ-permeability barrier function than Dvl1 or Dvl2 alone, and it was just as effective as Dvl1/2/3 triple knockdown. Each data point is a mean ± SE of *n* = 4 quadruple bicameral units of a representative experiment. *P < 0.05, when compared to the corresponding control by Student’s *t*-test. This experiment was repeated thrice with *n* *=* 3 independent experiments and yielded similar results

**Fig. 5:**
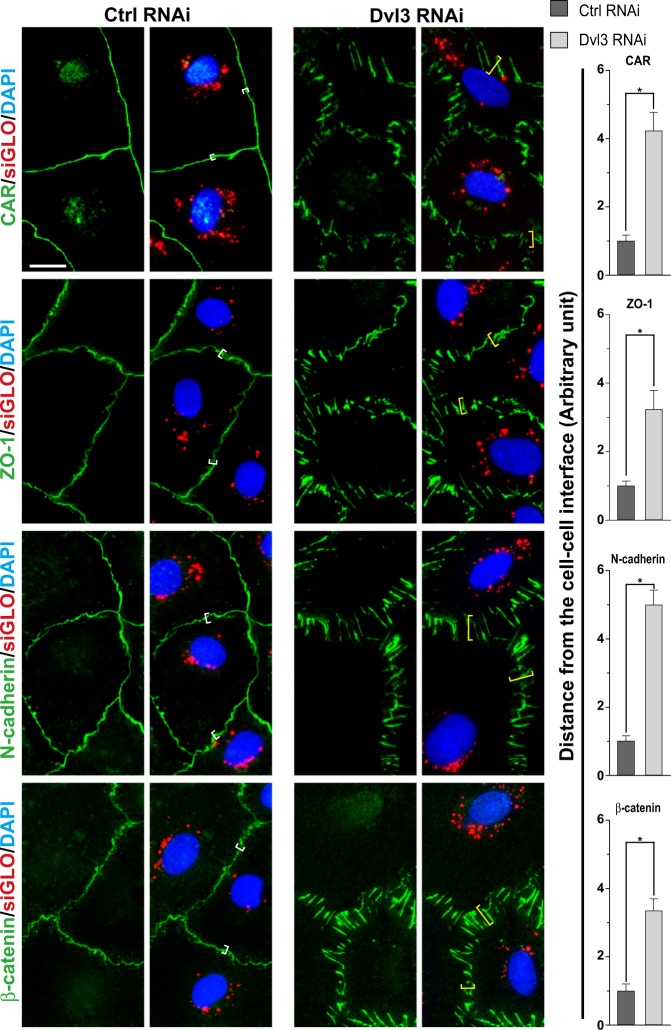
A knockdown of Dvl3 in Sertoli cells cultured in vitro induces changes in the distribution of BTB-associated proteins at the Sertoli cell–cell interface. A knockdown of Dvl3 by RNAi (Dvl3 RNAi) using specific siRNA duplexes vs. non-targeting negative control siRNA duplexes (Ctrl RNAi) was found to considerably perturb the distribution of TJ proteins (green fluorescence) CAR (an integral membrane protein) and ZO-1 (a TJ adaptor protein), and basal ES proteins (green fluorescence) N-cadherin (an integral membrane protein) and β-catenin (a basal ES adaptor protein). These proteins were localized at the Sertoli cell cortical zone in control cells (see white brackets). However, following Dvl3 knockdown by ~ 80% by RNAi, these proteins were rapidly endocytosed, no longer tightly localized at the Sertoli cell–cell interface (see yellow brackets) to confer the TJ-permeability barrier. These changes thus led to a loss of TJ-barrier function as noted in Fig. 4e. See Figure S6 for negative control IF images, illustrating the staining shown herein was specific to the corresponding marker protein. Scale bar, 40 µm, which applies to all other micrographs. Bar graphs on the right panel is the composite data in which each bar is a mean ± SD of *n* *=* 3 independent experiments using different batches of Sertoli cells, which yielded similar results. About 100 Sertoli cells from an experiment were randomly scored for analysis. *, *P* < 0.01 by Student’s *t* test when compared with the corresponding control group. Sertoli cell nuclei were stained with DAPI (blue fluorescence), and siGLO (red fluorescence) transfection indicator illustrates successful transfection

Primary Sertoli cells isolated from rat testes were used as a study model to monitor the TJ-permeability barrier function, and for RT-PCR, IB, and IF to assess changes in protein distribution at the Sertoli cell BTB using the regimen shown in Fig. 4a. As noted in Fig. 4b, knockdown of either Dvl1, 2, or 3 using specific siRNA duplexes by RNAi (Table 3), only the corresponding target gene was silenced without affecting the expression of the other two genes, illustrating the knockdown specificity. This notion was also supported by IB results in which changes in the steady-state levels of several PCP proteins (e.g., Fzd3, INVS, Vangl2), TJ proteins (CAR, ZO-1), basal ES proteins (N-cadherin, β-catenin), actin regulatory proteins (e.g., Eps8, formin1, Arp3), and MT-regulatory proteins (e.g., MARK4, EB1, Dia1, CLIP170) were assessed (Fig. 4c). A knockdown of Dvl3 by 80% by using Dvl3-specific siRNA duplexes vs. non-targeting control duplexes (Table 3) with the Sertoli cell lysates analyzed by IB, the steady-state levels of Dvl1 and Dvl2 were unaffected, illustrating the knockdown specificity (Fig. 4c). Furthermore, virtually all the examined proteins were not affected, except a considerably downregulation in Eps8 (an actin barbed end capping and bundling protein^37^ in the testis^38^) and an upregulation in MARK4 (MT affinity-regulating kinase 4, a Ser/Thr protein kinase known to induce phosphorylation of microtubule-associated proteins (MAPs) (e.g., MAP1, MAP2), causing detachment of MAPs from MTs, leading to MT catastrophe (see bar graphs on the right panel vs. the IB results on the left panel in Fig. 4c) were noted. These findings thus support the notion that the knockdown of Dvl3 is specific to this PCP protein, without any off-target effects. However, these changes in Eps8 and MARK4 expression illustrated that Dvl3 knockdown would possibly perturb actin organization (by downregulating Eps8) and MT stability (by upregulating MARK4). In order to further confirm the knockdown of Dvl3 in Sertoli cells, the expression of Dvl3 in the Sertoli cell epithelium was semi-quantitatively assessed by IF (see left and right panels in Fig. 4d). It was noted that silencing of Dvl3 by RNAi down-regulated the Dvl3 fluorescence signal by as much as 80% (Fig. 4d), consistent with IB data shown in Fig. 4c. More important, Dvl3 RNAi was effective to perturb the Sertoli cell TJ-barrier function, considerably more effective than either Dvl1 or Dvl2 knockdown by RNAi and similar to the results obtained by Dvl1/2/3 triple knockdown (Fig. 4e). In order to understand the mechanism by which Dvl3 knockdown that perturbed the Sertoli cell TJ-barrier function as noted in Fig. 4e, we examined if there were any changes in the distribution of TJ proteins CAR (an integral membrane TJ protein) and ZO-1 (a TJ adaptor protein) vs. basal ES proteins N-cadherin (an integral membrane protein) and β-catenin (an adaptor protein) (Fig. 5). As shown in Fig. 5, distribution of the TJ (CAR/ZO-1) and basal ES (N-cadherin/β-catenin) adhesion protein complexes at the Sertoli cell–cell interface following Dvl3 knockdown was considerably disrupted, in which these proteins no longer tightly distributed at the cell cortical zone, but internalized (Fig. 5, right panel).

### Dvl3 knockdown in Sertoli cells perturbs F-actin dynamics

**Fig. 6:**
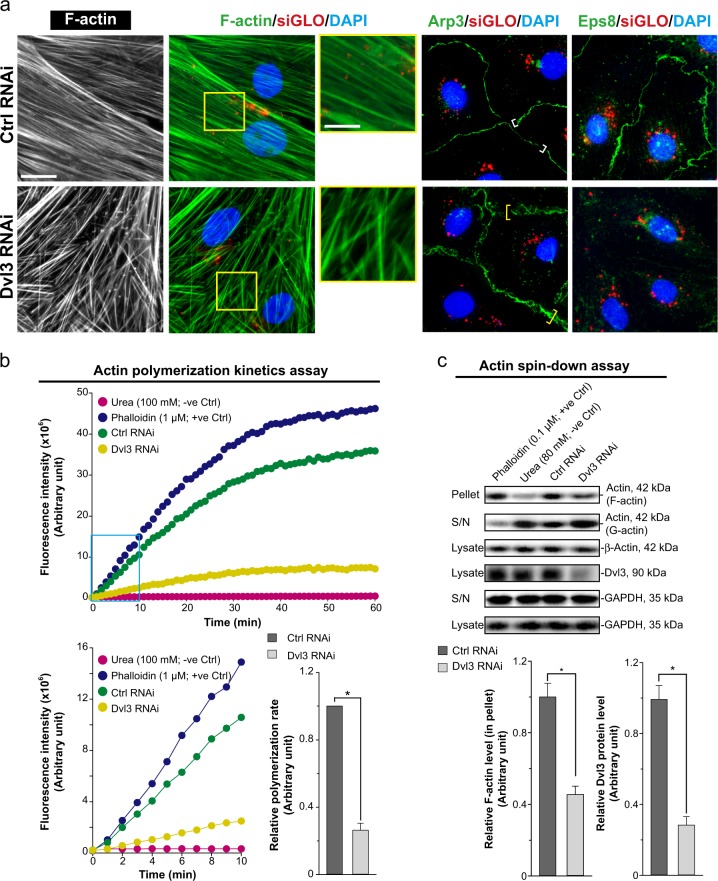
A knockdown of Dvl3 in Sertoli cells cultured in vitro perturbs the actin-based cytoskeletal organization and function. **a** In control cells transfected with non-targeting negative control siRNA duplexes (Ctrl RNAi), F-actin stretched across the Sertoli cell cytosol as linear bundles. However, following Dvl3 knockdown, actin filaments were extensively truncated and mis-organized, appearing as considerably branched filaments, which are clearly noted in the enlarged images boxed in yellow. These changes appeared to be the result of changes in spatial expression of Arp3 and Eps8 as these actin regulatory proteins no longer tightly localized at the Sertoli cell–cell interface to confer the Sertoli cell TJ-barrier function but endocytosed to Sertoli cell cytosol. For instance, Arp3 tightly associated at the Sertoli cell–cell interface in controls (see white brackets) were diffusely localized in Dvl3 silenced Sertoli cells (see yellow brackets). Sertoli cell nuclei were visualized by DAPI (blue fluorescence). See Figure S6 for negative control IF images, illustrating the staining shown herein was specific to the corresponding marker protein. Scale bar, 40 µm, which applies to corresponding micrographs in this panel; inset, 20 µm, which applies to other insets. **b** Findings of a representative biochemical assay, illustrating changes in actin polymerization kinetics following a knockdown of Dvl3, which impeded the ability of Sertoli cells to polymerize actin into linear filaments. This experiment was repeated with *n* *=* 3 using different batches of Sertoli cells, which yielded similar observations. In brief, polymerization of fluorescent pyrene-labeled actin was monitored by enhanced fluorescence emission at 395–440 nm over time (top panel). Changes in polymerization during the first 10 min, illustrating the rate of actin polymerization (increase in fluorescence intensity over time) during the initial linear phase in the first 10 min was plotted (lower left panel) and estimated by linear regression (lower right panel) to show the relative polymerization rate in Dvl3 RNAi vs. non-targeting negative control group. Each bar is a mean ± SD of *n* *=* 3 experiments. *, *P* < 0.01 compared with control RNAi by Student’s *t* test. **c** An actin-spin-down assay to monitor the relative levels of F- vs. G-actin, illustrating the ability of the Sertoli cell lysates to maintain polymerized actin filaments in these cell cultures. Dvl3 RNAi considerably perturbed the level of F-actin in cell lysates. These findings were results of a representative experiment of *n* *=* 3 experiments, and the composite data are shown in the lower panel. Uncropped IBs were shown in Figure S3. Histogram is the composite data wherein each bar is a mean ± SD of *n* = 3 experiments. *, *P* < 0.01 when compared with corresponding control by Student’s *t*-test

We next examined the molecular mechanism by which Dvl3 knockdown by RNAi that perturbed Sertoli cell TJ-barrier function (Fig. 4e) through disruptive changes in the distribution of TJ and basal ES adhesion protein complexes (Fig. 5). As noted in Fig. 6a, Dvl3 knockdown led to disruptive changes in F-action organization in Sertoli cells as actin filaments no longer stretched across the cell cytosol as found in controls, but considerably truncated and branched. These changes of actin-based cytoskeletal organization were possibly resulted from disruptive distribution and spatial expression of Arp3 and Eps8 as noted in Fig. 6a. Furthermore, based on the use of a biochemical assay that monitored actin polymerization including the appropriate negative  (urea) and positive (phalloidin) controls, Dvl3 RNAi considerably perturbed actin polymerization, as well as the kinetics of actin polymerization (Fig. 6b). These findings are also consistent with the data obtained by assessing the ability of Sertoli cell lysates to polymerize actin monomers to form F-actin in a spin-down assay (Fig. 6c). The findings shown herein that Dvl3 knockdown perturbed F-actin organization (Fig. 6) thus support the observation noted in Fig. 4b, regarding the considerably downregulation of Eps8, which is known to promote actin filaments to assume a bundled configuration, a downregulation of Eps8 thus favors actin cytoskeletal disorganization.

### Dvl3 knockdown in Sertoli cells perturbs MT dynamics

**Fig. 7:**
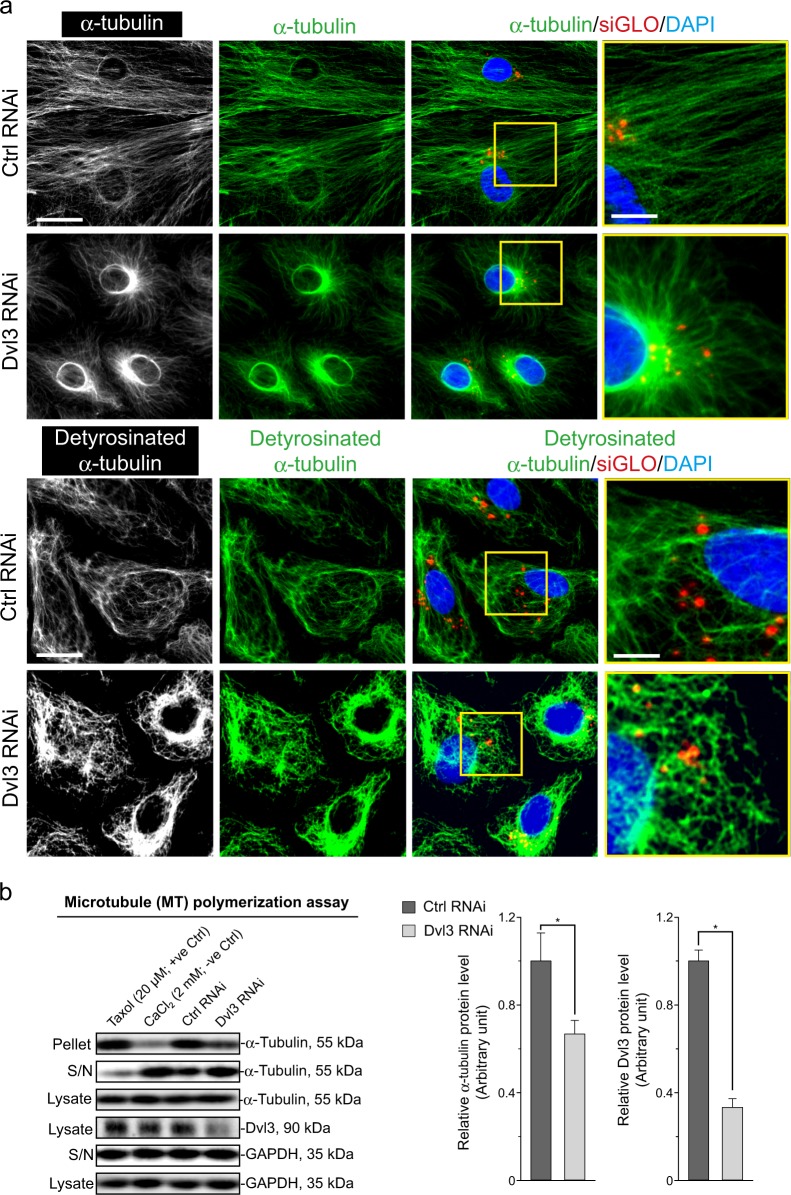
A knockdown of Dvl3 in Sertoli cells cultured in vitro perturbs the MT-based cytoskeletal organization and function. **a** In control cells transfected with non-targeting negative control siRNA duplexes (Ctrl RNAi), MTs (visualized by α-tubulin staining, green fluorescence, which together with β-tubulin create the α-/ß-tubulin dimers, which are the building blocks of MTs) stretched across the Sertoli cell cytosol. However, following Dvl3 knockdown, MTs were retracted from cell peripheries and stayed closer to the Sertoli cell nuclei. Sertoli cell nuclei were visualized by DAPI (blue fluorescence). The lower panel illustrates changes in the organization of detyrosinated α-tubulin, the more stabilized forms of α-tubulin^39^, as in control cells, detyrosinated α-tubulin appeared as linear while mildly branched MTs; however, after Dvl3 knockdown, α-tubulin appeared as extensively branched/truncated network (see magnified image in yellow boxed area). See Figure S6 for negative control IF images, illustrating the staining shown herein was specific to the corresponding marker protein. Scale bar, 40 µm, which applies to corresponding micrographs in this panel; 15 µm in boxed inset, which apply to corresponding insets. **b** Results of a representative biochemical assay that monitored the ability of Sertoli cell lysates to polymerize tubulins into MTs, which were recovered in the pellet vs. free tubules in S/N (supernatant). It was noted that a knockdown of Dvl3 by RNAi perturbed the ability of the Sertoli cells to polymerize MTs considerably. Uncropped IBs were shown in Figure S4. The histogram shown on the right panel is the composite data of *n* = 3 independent experiments using different batches of Sertoli cells following knockdown of Dvl3 with each bar a mean ± SD of three experiments, and each experiment had triplicate cultures. *, *P* < 0.05 when compared with the corresponding control by Student’s *t* test

As noted in Fig. 7a, Dvl3 knockdown by RNAi also perturbed the organization of MTs in Sertoli cells wherein α-tubulin (a building block of MTs) no longer stretched across the entire Sertoli cell cytosol but retracted from cell peripheries and moved closer to the cell nuclei (Fig. 7a). These changes noted in MT organization in Fig. 7a possibly owing to defects in MT polymerization following Dvl3 knockdown. This possibility, indeed, was confirmed based on a biochemical study by assessing MT polymerization using lysates from Dvl3 silenced Sertoli cells vs. control cells as there was a considerably reduction in MT polymerization following Dvl3 knockdown (Fig. 7b). Interestingly, we also noted that the distribution of detyrosinated α-tubulin (the stabilized form of α-tubulins^39^) across the Sertoli cell was considerably disrupted since detyrosinated α-tubulin no longer stretched across the cell cytosol as the typical MT protofilaments, but appeared as aggregated protofilaments (Fig. 7a). These changes in MT organization, perhaps, were mediated by an upregulation of MARK4 as shown in Fig. 4c since MARK4 is known to induce MT catastrophe^40^, leading to MT disorganization as noted in Fig. 7a.

### Dvl1/2/3 triple knockdown in the testis in vivo induces defects in spermatogenesis

**Fig. 8:**
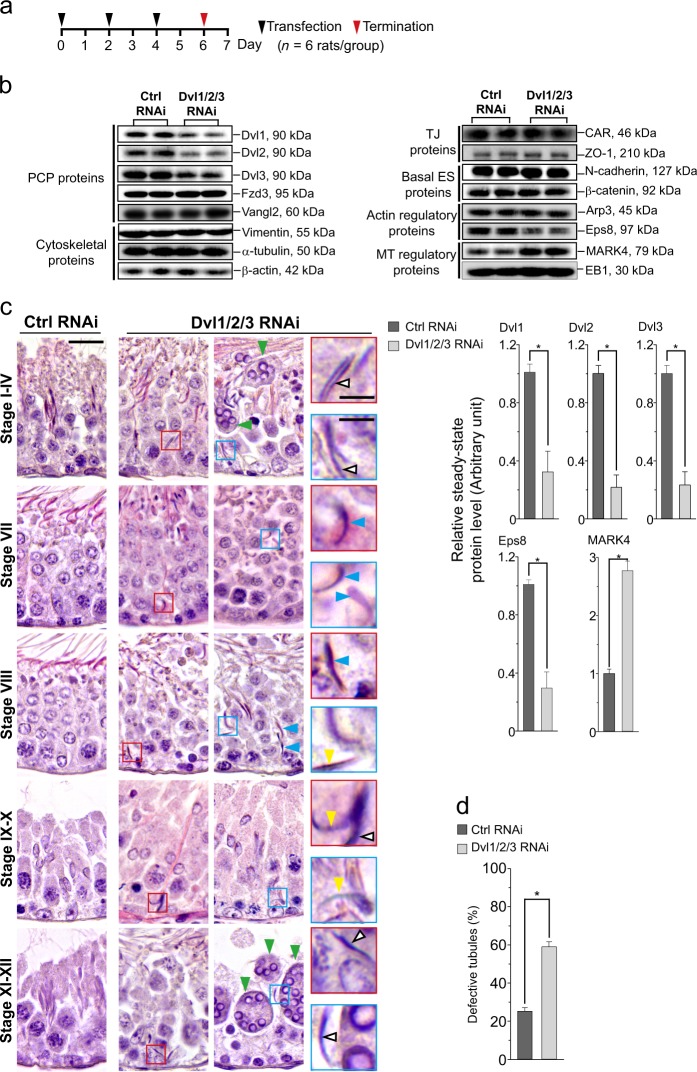
A knockdown of Dvl1/2/3 in the testis in vivo perturbs spermatogenesis. **a** Regimen used to treat adult rats with *n* = 6 rats per treatment or control group. Testes received the Dvl1, Dvl2, and Dvl3-specific siRNA duplexes (triple knockdown) vs. non-targeting negative control siRNA duplexes on day 0, 2, and 4 (thrice) and rats were killed on day 6 for either IB, histological analysis, or immunofluorescence (IF) analysis. This regimen was established based on results of pilot experiments as a knockdown of Dvl3 alone failed to induce considerable phenotype in the testis, thus a triple knockdown by RNAi was used instead. **b** Representative IB data from *n* = 3 experiments, illustrating successful knockdown of Dvl1, Dvl2, and Dvl3, without affecting the expression of other PCP proteins such as Fzd3 and Vangl2, nor other BTB-associated except for a considerable downregulation of actin barbed end capping and bundling protein Eps8 (known to maintain actin filament bundles at the ES), and upregulation of MARK2 regulatory protein (known to promote MT catastrophe, i.e., destabilizing MTs), consistent with findings in vitro (see Fig. 4c). Uncropped IBs were shown in Figure S5. Histograms in the lower panel are the composite IB data such as those shown in the upper panel from *n* = 3 independent experiments. Each bar is a mean ± SD of three experiments. *, *P* < 0.01 when compared with the corresponding control by Student’s *t* test. **c** Histological analysis was performed using paraffin sections (~ 5 µm thick) of testes (fixed in modified Davidson fixative) and stained with hematoxylin and eosin. Control testes transfected with non-targeting negative control siRNA duplexes had normal spermatogenesis in the seminiferous epithelium (left panel). However, considerable defects in spermatogenesis were detected in the epithelium from testes following Dvl1/2/3 knockdown by RNAi as noted in the second and third columns; selected areas in these two columns were boxed in either red or blue and magnified in the corresponding insets to illustrate defects. These include: (i) step 19 spermatids (white arrowheads) found in stage I–IV tubules, co-existing with steps 15–17 spermatids, and also some multinucleated round spermatids (green arrowheads); (ii) step 19 spermatids remained trapped deep inside the epithelium in stage VII or VIII tubules (blue arrowheads) when they should have been transported near the tubule lumen to prepare for their release at spermiation, also defects in spermatid polarity were noted with these spermatids no longer pointed toward the basement membrane but deviated by 90°–180° from the intended orientation (yellow arrowheads); (iii) step 19 spermatids were detected in the epithelium, co-existing with step 9,10, 11, or 12 spermatids (white arrowheads) and mult-nucleated round spermatids (green arrowheads) in stages IX–X and XI–XII tubules, and some step 19 spermatids had defects in polarity (yellow arrowhead); and (iv) considerable thinning of the epithelium was also detected, possibly due to unwanted release of elongated spermatids as a result of defects in cytoskeletal organization. Scale bar, 80 µm in first panel, which applies to all other images in first, second and third panels; 40 µm in red and blue insets, which apply to all corresponding insets. **d** Percentage (%) of defective tubules from at least 100 randomly scored tubules from cross-sections of a rat testis with *n* = 4 rats. *, *P* < 0.01 compared with the control group by Student’s *t* test

For in vivo studies, we opted to knockdown Dvl1/2/3 (triple knockdown) in the testis since pilot experiments using *n* = 3 rats had shown that defects of spermatogenesis in seminiferous tubules had limited to just ~ 20% when only Dvl3 was silenced by ~80%. In light of the data shown in Fig. 4b, c that the siRNA duplexes specific to Dvl1 and Dvl2 were effective to induce specific knockdown of the corresponding Dvl gene, we limited this study of a triple knockdown of Dvl1/2/3. Figure 8a illustrates the regimen used for the in vivo studies involving three transfection on day 0, 2, and 4 with the termination performed on day 6 with *n* = 6 rats for the treatment and also for the control groups. As noted in Fig. 8b, when Dvl1, 2, and 3 was silenced by ~ 70–80% based on immunoblot analysis (see bar graphs in the lower panel of Fig. 8b) of *n* = 3 experiments, none of the examined proteins were affected except for Eps8 and MARK4 which was down- or upregulated, respectively (see also bar graph in the lower panel in Fig. 8b), consistent with in vitro data shown in Fig. 4c. Based on histological analysis, multiple defects in spermatogenesis were noted in the seminiferous epithelium following Dvl1/2/3 knockdown vs. control testes (Ctrl RNAi). These include: (i) appearance of multinucleated round spermatids (green arrowheads) which are haploid spermatids to be phagocytosed by Sertoli cells for degradation, (ii) step 19 spermatids trapped deep inside the epithelium, failing to be transported to near the tubule lumen to be emptied into the tubule lumen at spermiation (blue arrowheads), (iii) some of these step 19 spermatids persisted in stage IX–X and XI–XII tubules (white arrowheads), co-existing with step 9, 10, 11, and 12 spermatids, (iv) defects of spermatid polarity in which their heads failed to point toward the basement membrane but deviated by 90°–180° from their intended orientation (yellow arrowheads) as noted in control testes (Fig. 8c). More important, when Dvl1/2/3 was silenced by RNAi, > 60% of the tubules displayed defects in spermatogenesis (Fig. 8d).

### Dvl1/2/3 triple knockdown perturbs spermatogenic function through disruptive changes in actin and MT dynamics

**Fig. 9:**
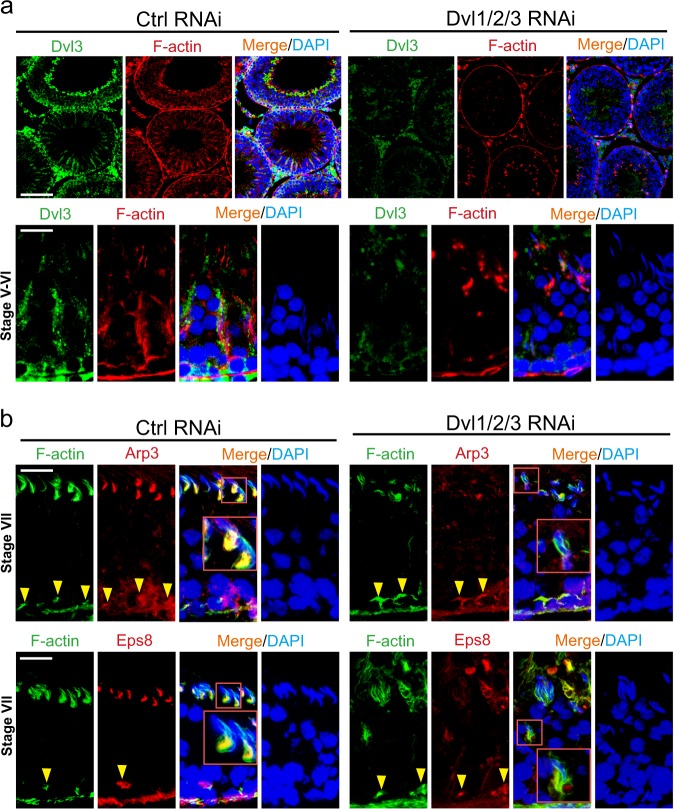
Dvl1/2/3 triple knockdown in the testis in vivo perturbs spermatogenesis through disruptive changes in actin cytoskeletal organization by altering the spatiotemporal expression of actin regulatory proteins Arp3 and Eps8. **a** Distribution of Dvl3 (green fluorescence) and its co-localization with F-actin (red fluorescence) in the seminiferous epithelium in control testes noted herein were similar to findings shown in Figs 1e and 2a, wherein Dvl3 appeared as track-like structures co-localized with F-actin in stage V–VI tubules, associated with elongating spermatids. However, the expression of Dvl3 across the seminiferous epithelium was considerably diminished following Dvl1/2/3 knockdown by RNAi in the testis. The disruptive changes in the organization of F-actin across the seminiferous epithelium in tubules (such as stage V–VI tubules) were remarkable in magnified images shown in the lower panel following a knockdown of Dvl1/2/3 as illustrated by Dvl3 (green fluorescence) immunofluorescence staining. For instance, F-actin no longer highly expressed at the apical ES and orderly aligned across the seminiferous epithelium to support elongating/elongated spermatids. Scale bar, 350 µm and 80 µm, in the top and bottom panel, respectively, which apply to other images in the corresponding panel. **b** In control testes, Arp3 (red fluorescence, an branched actin polymerization protein, causing linear actin filaments to assume a branched configuration) or Eps8 (red fluorescence, an actin barbed end capping and bundling protein, causing linear actin filaments to assemble into bundles as noted in the ES) and F-actin (green fluorescence) were predominantly expressed and localized (and also co-localized) at the concave (ventral) side of elongated spermatid heads (see enlarged images in red boxed areas), and also at the basal ES/BTB (annotated by yellow arrowheads) as noted in the corresponding upper and lower panels in stage VII tubules. However, following knockdown of Dvl1/2/3 by RNAi, Arp3, and Eps8 no longer expressed prominently at the apical ES, but considerably downregulated and no longer restrictively expressed at the concave side of spermatid heads (see corresponding enlarged images in red boxed areas). It appeared that Dvl1/2/3 knockdown did not considerably  affect spatial expression of Arp3 or Eps8, nor distribution of F-actin, at the basal ES/BTB. Scale bar, 80 µm, which applies to corresponding micrographs in the same panel. See Figure S7 for negative control IF images, illustrating the staining shown herein was specific to the corresponding marker protein

**Fig. 10:**
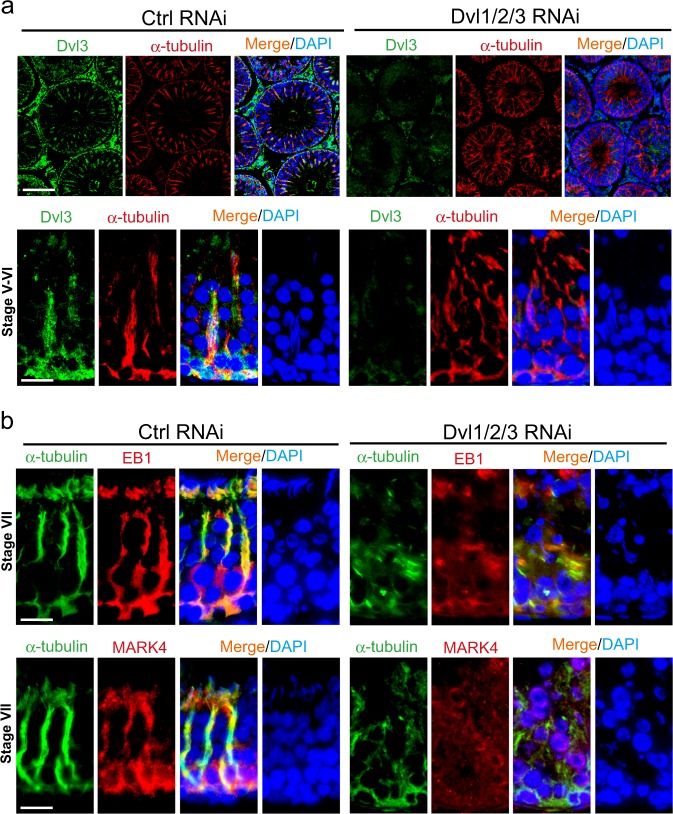
Dvl1/2/3 triple knockdown in the testis in vivo perturbs spermatogenesis through disruptive changes in MT cytoskeletal organization by altering the spatiotemporal expression of MT-regulatory proteins EB1 and MARK4. **a** Distribution of Dvl3 (green fluorescence) and its colocalization with α-tubulin (red fluorescence, which together with β-tubulin that create the α-/β-tubulin dimers are the building blocks of MTs) in the seminiferous epithelium in control testes noted herein were similar to findings shown in Fig. 2b, wherein Dvl3 appeared as track-like structures colocalized with MTs in stage V–VI tubules, associated with elongating spermatids. However, the expression of Dvl3 across the seminiferous epithelium was considerably diminished following Dvl1/2/3 knockdown by RNAi in the testis. The disruptive changes in the organization of MTs across the seminiferous epithelium in tubules (such as stage V–VI tubules) were remarkable in magnified images shown in the lower panel following a knockdown of Dvl1/2/3 as illustrated by Dvl3 immunofluorescence staining. For instance, the track-like structures conferred by MTs that laid perpendicular to the basement membrane were extensively truncated, no longer extended across the entire seminiferous epithelium. Scale bar, 350 µm and 80 µm, in the top and bottom panel, respectively, which apply to other images in the corresponding panel. **b** In control testes, EB1 (red fluorescence, a + TIP protein that is known to stabilize MTs^43^) or MARK4 (red fluorescence, a Ser/Thr protein kinase known to phosphorylate MAPs (e.g., MAP1a), causing their dissociation from MTs, thereby leading to MT catastrophe^68, 71^) and α-tubulin (green fluorescence) were predominantly expressed and localized (and also colocalized) at the concave (ventral) side of elongated spermatid heads, and also the track-like structures across the epithelium as noted in the corresponding upper and lower panels in stage VII tubules. However, following knockdown of Dvl1/2/3 by RNAi, EB1, and MARK4 no longer expressed prominently at the apical ES (and fewer elongated spermatids were detected in the seminiferous epithelium) and across the seminiferous epithelium, but considerably down-regulated. Scale bar, 80 µm, which applies to corresponding micrographs in the same panel. See Figure S7 for negative control IF images, illustrating the staining shown herein was specific to the corresponding marker protein

Following Dvl1/2/3 knockdown in the testis, there was a considerable reduction in Dvl3 (green fluorescence) staining across the seminiferous epithelium as noted in Fig. 9a in both low and high magnified images (see upper and lower panel in Fig. 9a), supporting the notion of the considerable knockdown of Dvl genes. Consistent with the in vitro data earlier shown in Fig. 6 that Dvl3 knockdown impeded F-actin organization in Sertoli cells, Dvl1/2/3 knockdown also perturbed the organization of F-actin (red fluorescence) across the seminiferous epithelium (Fig. 9a). This disruptive changes in the organization of F-actin (green fluorescence) was likely the result of changes in the spatial expression of branched actin polymerization protein Arp3 (red fluorescence) and Eps8 (red fluorescence) (Fig. 9b). For instance, Apr3 and Eps8 appeared as bulb-like structures at the concave (ventral) side of spermatid head in control testes, but after Dvl1/2/3 knockdown, the expression of these two proteins was considerably diminished and mis-localized even though their distribution at the basal ES/BTB was not considerably affected (see yellow arrowheads in Fig. 9b). Similar to changes in F-actin organization, a knockdown of Dvl1/2/3 in the testis also perturbed MT organization as seen through staining of α-tubulin (which together with β-tubulin that create the α-/β-tubulin dimers are the building blocks of MTs^41,42^) (Fig. 10a). For instance, the MT-conferred tracks that laid perpendicular to the basement membrane that stretched across the entire seminiferous epithelium were extensively truncated (Fig. 10a). More importantly, these disruptive changes in MT organization apparently noted in Fig. 10a were results of changes in spatial expression of EB1 (a + TIP protein known to induce MT stability^32,43,44^) and MARK4 (a Thr/Ser protein kinase known to induce MT catastrophe^40,45^) (Fig. 10b). The distribution of either EB1 or MARK4 was grossly perturbed as none of these proteins colocalized with α-tubulin and stretched across the entire epithelium but extensively truncated (Fig. 10b), thereby failing to support spermatid and organelle (e.g., phagosomes) transport across the epithelium during the epithelial cycle, leading to defects in spermatogenesis as noted in Fig. 8c.

## Discussion

Studies have shown that Dishevelled proteins, such as Dvl1, Dvl2, and Dvl3, act as adaptors (or scaffold proteins) that are working in partnership with Fzd (an integral membrane protein) to create the PCP protein complex of Fzd/Dvl, which in turn induces Wnt signaling downstream, having a crucial role to support development, tissue regeneration, cellular homeostasis, and an array of other cellular processes^46–48^. However, the precise biological function of Dvl proteins remains a mystery after more than two decades of studies^49^. Other studies have also shown that the Fzd/Dvl/Invs (Inversin,  another adaptor/scaffold partner protein) PCP complex modulates actin-based cytoskeletal function through Rho GTPase^11,50^. Nonetheless, studies using mouse genetics models have shown that Dvl1, Dvl2, and Dvl3 are crucial regulators of tissue/organ development, even though their role and/or involvement in testis function and spermatogenesis remain unexplored. For instance, although *Dvl1*^*−/−*^ mice appeared to be physiologically normal (i.e., mice were viable and fertile without developmental defects), these mice exhibited reduced social interactions and defects in behavioral wellness^51^. Subsequent study has shown that *Dvl1*^*−/−*^ mice also had defects in gastrointestinal (GI) tract owing to abnormalities in GI microbiota and immune CD8+ T cells in GI tract, illustrating its role in maintaining homeostasis of the intestine^52^. On the other hand, mice containing null mutation in *Dvl2* (*Dvl2*^*−/−*^) were found to have 50% lethality owing to severe cardiovascular outflow tract defects, with the majority of the surviving *Dvl2*^*−/−*^ mice were female, and 90% of the *Dvl2*^*−/−*^ mice were having vertebral and rib malformation^53^. However, *Dvl3*^*−/−*^ mice led to perinatal fatality owing to cardiac outflow tract abnormalities^54^, and as such, the role of *Dvl3* in testis and spermatogenic function remains unknown. In humans, mutations in *Dvl3*^55^ or *Dvl1*^56^ led to autosomal–dominant Robinow syndrome, characterized by mesomelic limb shortening, genital hypoplasia, and distinctive facial features. Whereas mutations of *Dvl2* and *Dvl1* in humans also led to inflammatory bowel disease^57,58^. In short, studies from using genetic models and/or mutation analysis in humans do not provide insightful information regarding functions of these Dvl proteins, including Dvl3, in testis and spermatogenesis  . As reported herein, a knockdown of Dvl1/2/3 in the testis in vivo indeed was shown to perturb spermatid polarity as many elongating/elongated spermatids in different stages of the epithelial cycle displayed defects in polarity whereby their heads no longer oriented directionally by pointing to the basement membrane but deviated by 90°–180° from the intended orientation. Besides defects in spermatid polarity, spermatid adhesion was also grossly affected as many spermatids had undergone spermiation in stages VI–VIII early tubules. Yet some step 19 spermatids remained deeply embedded into the epithelium, persistently found and intermingled with step 9–14 spermatids in stages IX–XIV tubules. Taking collectively, these findings suggest that a loss of Dvl1/2/3 function perturbs the actin- and perhaps MT-based cytoskeletons.

In studies in vitro, the actin filaments and MTs that laid across the Sertoli cell cytosol in Dvl3 silenced cells were grossly affected when compared with control cells, as they were either truncated or re-distributed following Dvl3 knockdown to be close to the cell nuclei. These morphological findings regarding actin and/or MT organization in Sertoli cells were confirmed when the ability of Sertoli cells to induce actin polymerization and also MT polymerization was assessed by biochemical assays. For instance, following Dvl3 knockdown, the kinetics of actin polymerization or the ability of Sertoli cell lysate to polymerize MTs were considerably diminished vs. control cells. Furthermore, these findings in vitro were also confirmed and expanded in studies in vivo. For instance, when Dvl1/2/3 were silenced in the testis in vivo, this caused disruptive changes on the organization of both F-actin and MTs across the seminiferous epithelium such that these track-like structures conferred by F-actin or MTs were no longer found in the epithelium, instead they were extensively truncated. These broken tracks no longer support spermatid adhesion at the apical ES which is the only anchoring device that maintain spermatid adhesion in the testis^59–62^. In addition, the disrupted actin- and/or MT-based tracks also failed to support the timely transport of spermatids across the epithelium during the epithelial cycle, leading to premature release of spermatids in non-stage VIII tubules, mimicking spermiation at stage VIII tubules. These disruptive effects on the actin- and MT-based cytoskeletons following Dvl1/2/3 knockdown in the testis are consistent with recent studies, reporting the role of Dvl proteins to maintain actin- and MT-based cytoskeletal function in multiple epithelia and/or tissues^63–66^. A recent report using a yeast-two hybrid screen has shown that Eps8 is a direct interactor of Dvl1^63^, and Eps8 silencing also blocks the axon remodeling activity of Wnt3a^63^. Taking collectively, these results thus illustrate that Eps8, an actin barbed end capping and bundling protein^37,67^ earlier shown to have a crucial role to support actin filament bundles at the ES^38^, may be involved in the Dvl protein function. Herein, we have shown that a knockdown of Dvl3 by RNAi downregulates Eps8 and upregulates MARK4 (a Ser/Thr protein kinase known to phosphorylate MAPs such as MAP1a, which in turn dissociates from MTs, leading to MT catastrophe^40,68^), respectively. These findings suggest that Dvl3 modulates actin dynamics by modifying actin filament and MT organization through the respective action of actin barbed end capping/bundling protein Eps8 and MT catastrophe-inducing protein MARK4. These findings also illustrate the PCP proteins Dishevelled are playing a very determining role in modulating cytoskeletal organization, at least in the testis, to support spermatogenesis.

In this context, it is of interest to note that even though a triple knockdown of Dvl1/2/3 by ~ 70–80% considerably perturbed spermatogenesis, wherein ~ 60% of the tubules where found to have various defects of spermatogenesis as reported here. This incomplete disruption on spermatogenesis is likely the result of transfection efficiency, which was estimated to be at ~ 70% when the Polyplus transfection reagent was used, and considerably better that the conventional liposome-based transfection medium at ~ 20–30%^69^. We elected not to perform standard fertility tests in these rats as studies have shown that when the spermatogenic output in rodents was disrupted by 90%, they remained fertility^70^. Nonetheless, we have provided compelling evidence that a knockdown of the Dishevelled family, most notably Dvl1/2/3, in the testis in vivo impeded spermatogenesis, through disruptive changes in the organization of actin- and MT-based cytoskeletons. These findings will provide sufficient information for future functional studies.

## Supplementary information

Supplemental Material
