# Supplementary material for: Planar cell polarity protein Dishevelled 3 (Dvl3) regulates ectoplasmic specialization (ES) dynamics in the testis through changes in cytoskeletal organization
Source: Cell Death Dis. 2019 Feb 26;10(3):194. doi: 10.1038/s41419-019-1394-7 (PMC6391420; doi:10.1038/s41419-019-1394-7)
Supplement: Supplementary file 1 — Supplemental Material [file 41419_2019_1394_MOESM1_ESM.pdf]

Figure S1 (Li et al.)

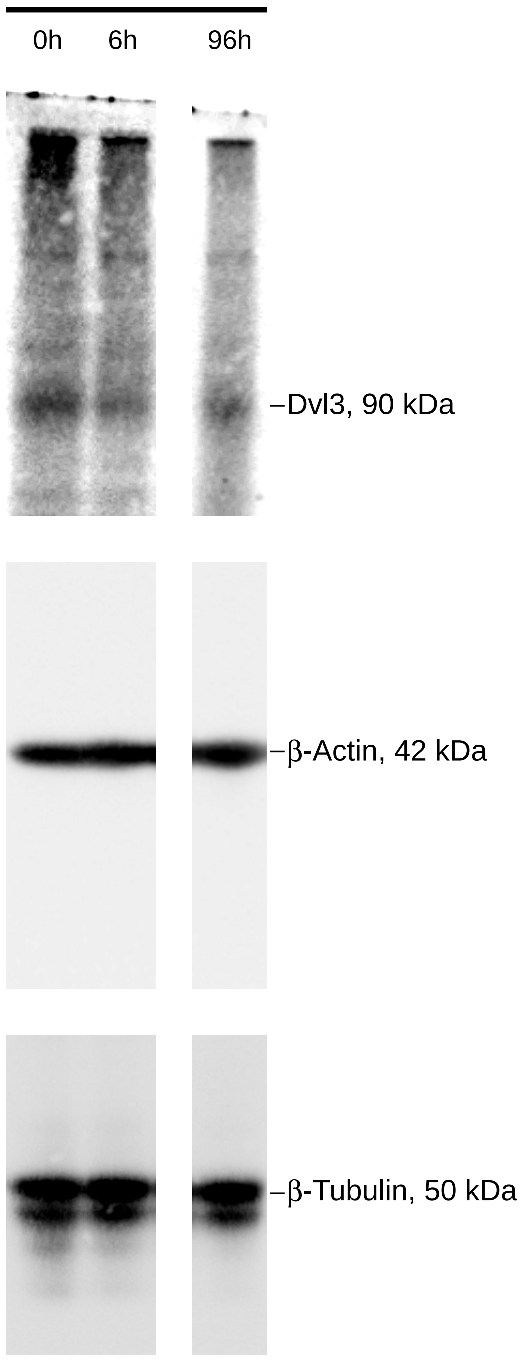

Figure S1. Uncropped immunoblotts corresponding to blots shown in Figure 3a.

Figure S2 (Li et al.)

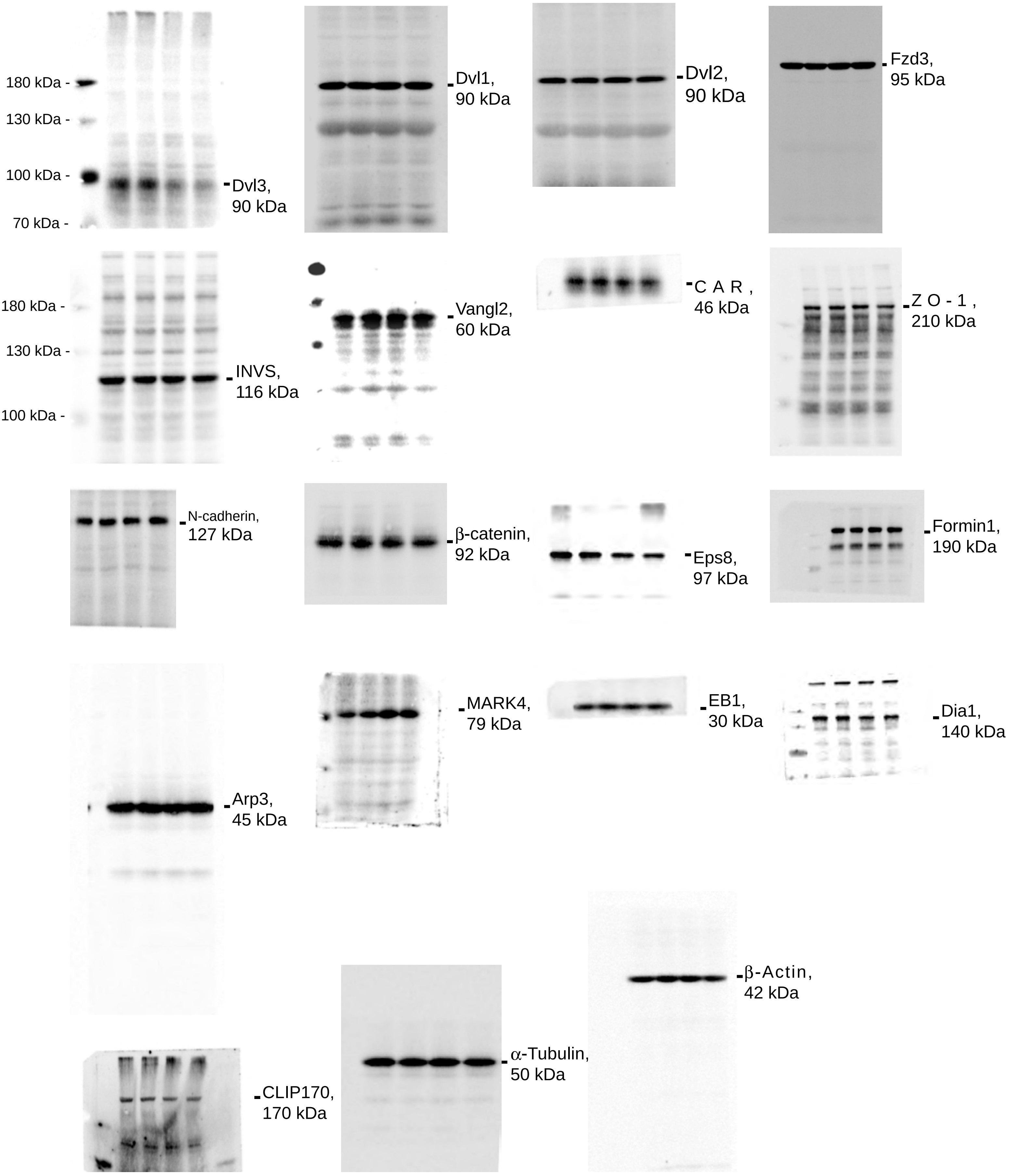

Figure S2. Uncropped immunoblotts corresponding to blots shown in Figure 4c.

Figure S3 (Li et al.)

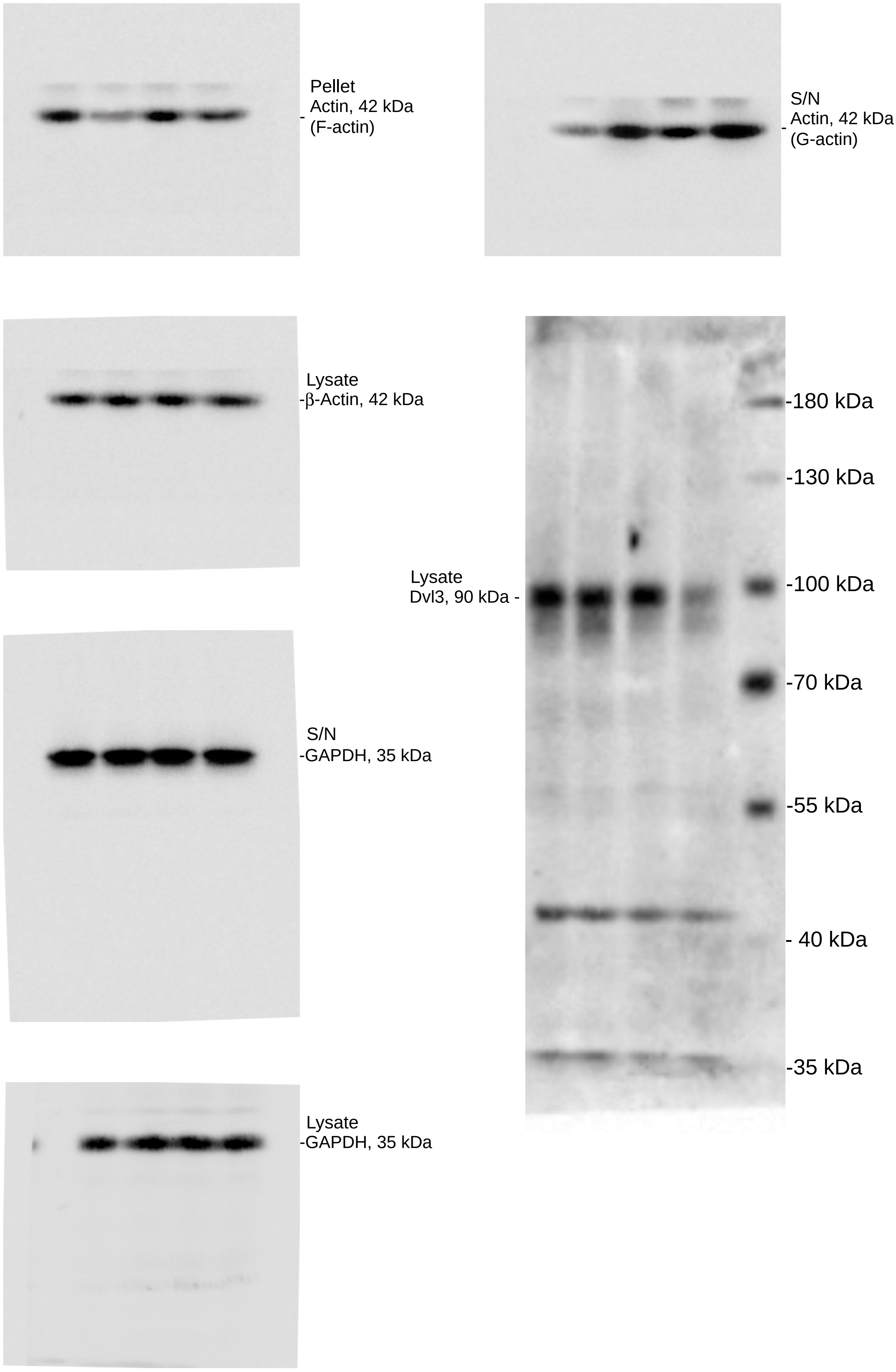

Figure S3. Uncropped immunoblotts corresponding to blots shown in Figure 6c.

Figure S4 (Li et al.)

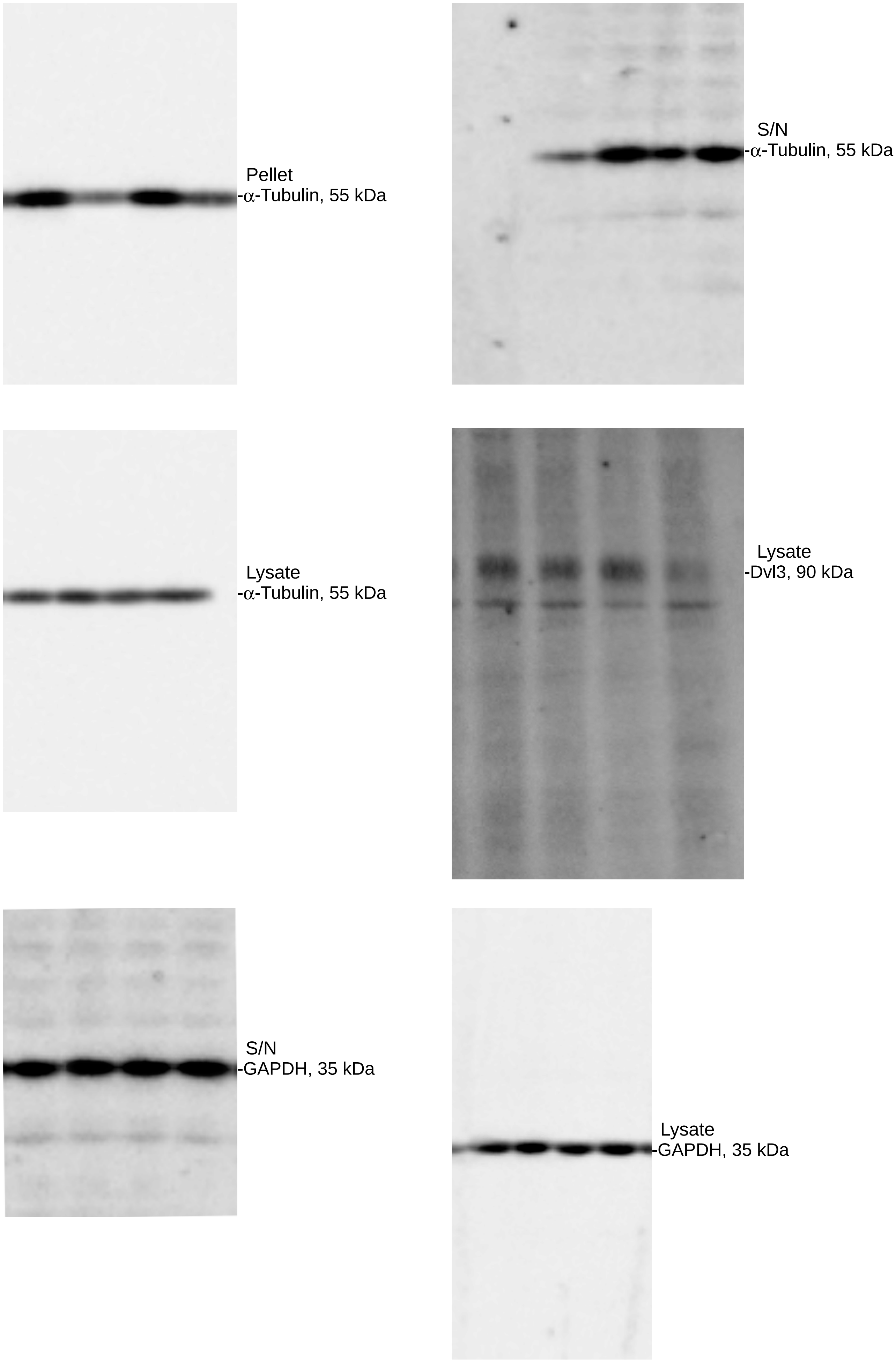

Figure S4. Uncropped immunoblotts corresponding to blots shown in Figure 7b.

Figure S5 (Li et al.)

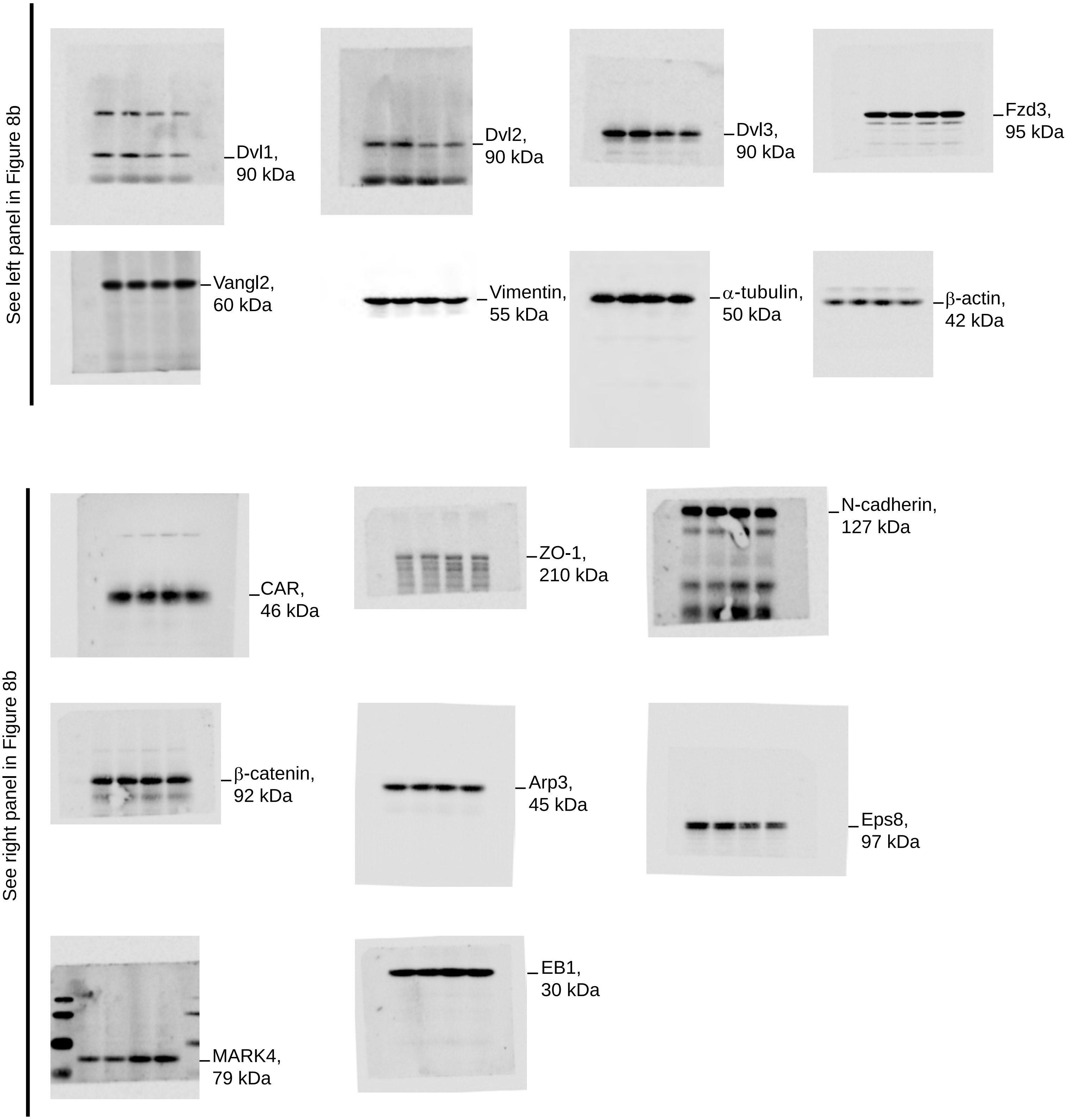

Figure S5. Uncropped immunoblotts corresponding to blots shown in Figure 8b.

Figure S6 (Li et al.)

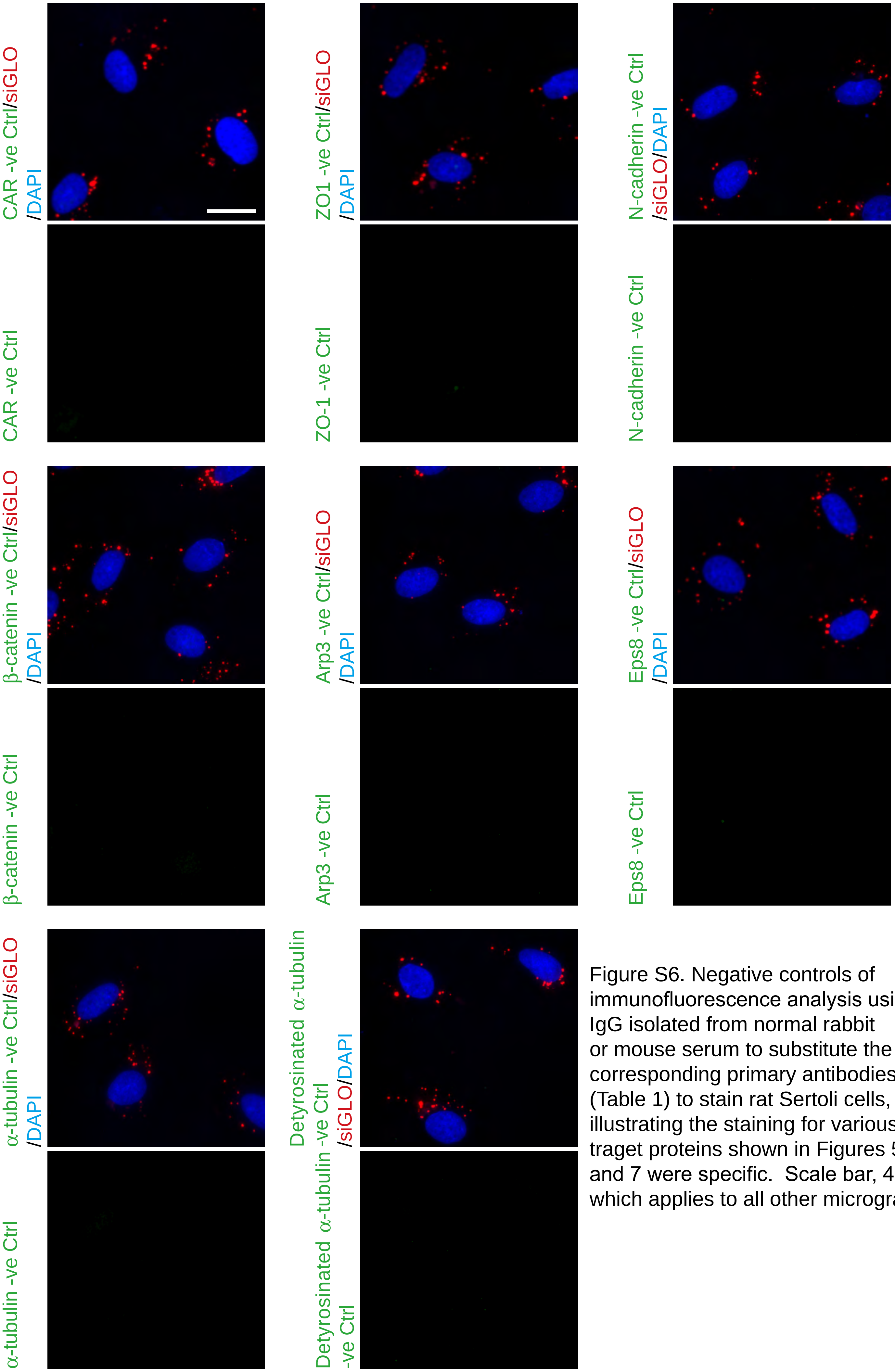

Figure S6. Negative controls of immunofluorescence analysis using IgG isolated from normal rabbit or mouse serum to substitute the corresponding primary antibodies (Table 1) to stain rat Sertoli cells, illustrating the staining for various traget proteins shown in Figures 5, 6 and 7 were specific. Scale bar, 40 μm which applies to all other micrographs.

Figure S7 (Li et al.)

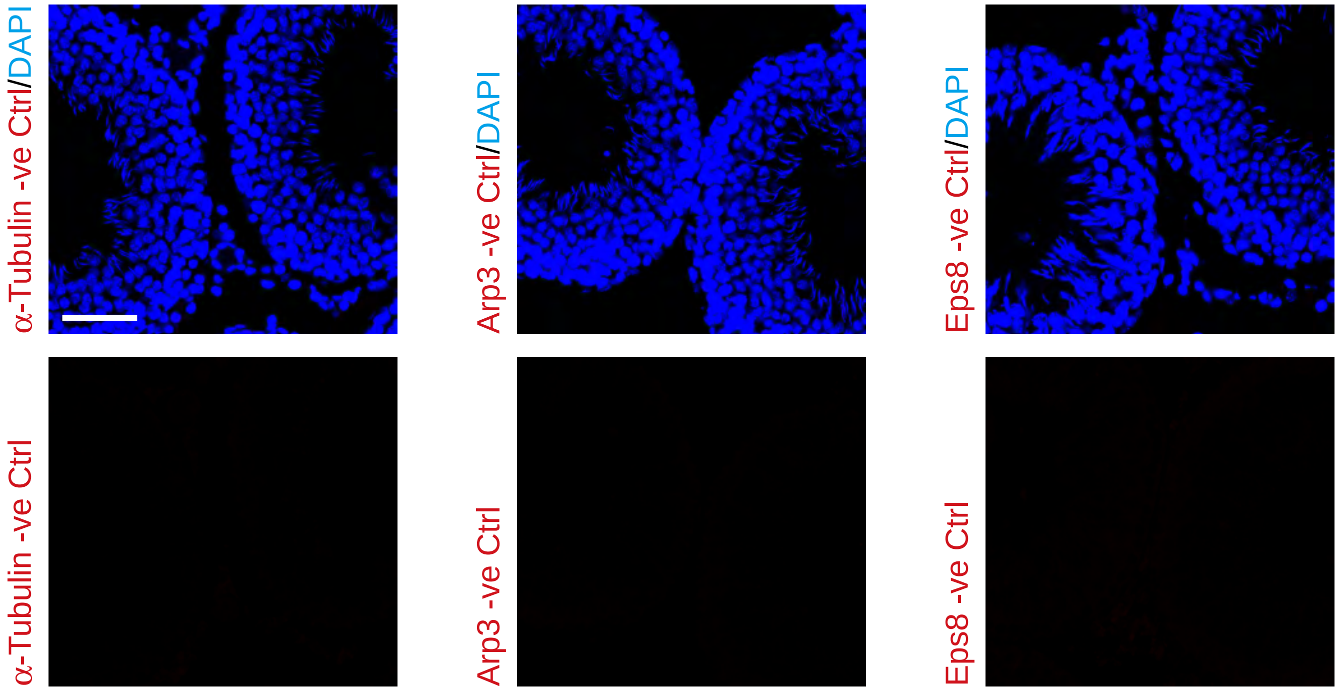

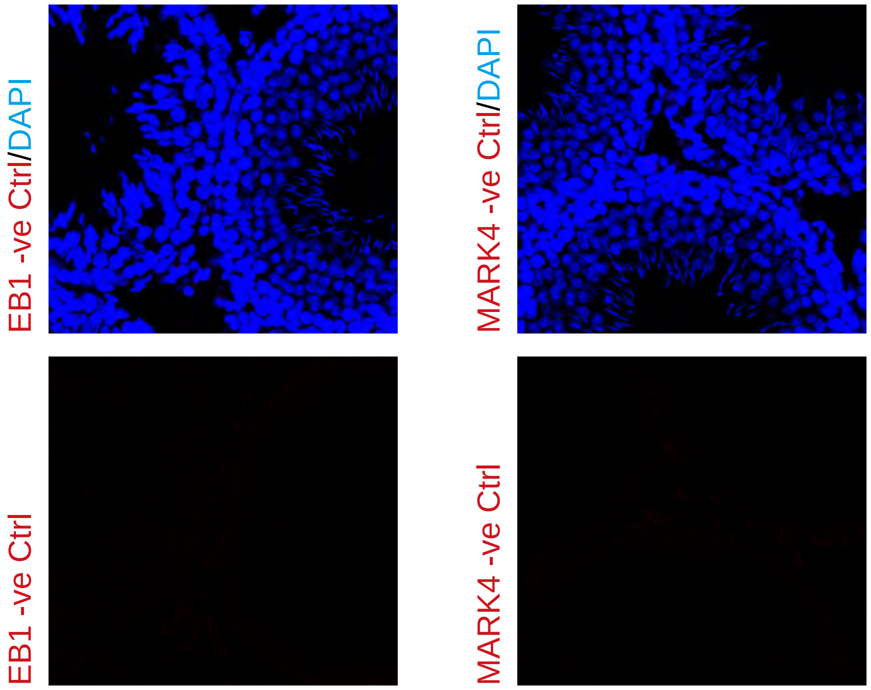

Figure S6. Negative controls of immunofluorescence analysis using IgG isolated from normal rabbit or mouse serum to substitute the corresponding primary antibodies (Table 1) to stain cross-sections of adult rat testes, illustrating the staining for various traget proteins shown in Figures 9 and 10 were specific. Scale bar, 300 µm which applies to all other micrographs.
